# A review of the Cholevinae from the island of Borneo (Coleoptera, Leiodidae)

**DOI:** 10.3897/zookeys.777.23212

**Published:** 2018-07-30

**Authors:** Menno Schilthuizen, Michel Perreau, Iva Njunjić

**Affiliations:** 1 Naturalis Biodiversity Center, Darwinweg 2, 2333CR Leiden, The Netherlands Naturalis Biodiversity Center Leiden Netherlands; 2 Institute for Biology Leiden, Leiden University, Sylviusweg 72, 2333BE Leiden, The Netherlands Leiden University Leiden Netherlands; 3 Taxon Expeditions, Rembrandtstraat 20, 2311 VW Leiden, The Netherlands Taxon Expeditions Leiden Netherlands; 4 Institute for Tropical Biology and Conservation, Universiti Malaysia Sabah, Jalan UMS, Kota Kinabalu, Malaysia Universiti Malaysia Sabah Kota Kinabalu Malaysia; 5 IUT Paris Diderot, Université Paris Diderot, Sorbonne Paris cité, case 7139, 5 rue Thomas Mann, 75205 Paris cedex 13, France Université Paris Diderot Paris France; 6 Institut de Systématique, Évolution et Biodiversité, Muséum National d’Histoire naturelle, Sorbonne Universités, Paris, France Muséum National d’Histoire naturelle, Sorbonne Universités Paris France

**Keywords:** caves, Indonesia, Malaysia, scavenging beetles, taxonomy

## Abstract

The available knowledge of the round fungus beetle subfamily Cholevinae (Leiodidae) from the island of Borneo is reviewed, and the results of newly studied material presented. The currently known 30 species (of which 14 are newly described herein) represent the genera *Micronemadus* (one species), *Catops* (one species), *Baryodirus* (one species), *Ptomaphaginus* (14 species), and *Ptomaphaminus* (13 species). The following new species are described: *Micronemadussondaicus* Schilthuizen & Perreau, **sp. n.**, *Ptomaphaginusgrandis* Schilthuizen & Perreau, **sp. n.**, *P.louis* Schilthuizen & Perreau, **sp. n.**, *P.muluensis* Schilthuizen & Perreau, **sp. n.**, and *P.isabellarossellini* Schilthuizen, Njunjić & Perreau, **sp. n.**, and *Ptomaphaminuskinabatanganensis* Njunjić, Schilthuizen & Perreau, **sp. n.**, *P.testaceus* Schilthuizen & Perreau, **sp. n.**, *P.nanus* Schilthuizen & Perreau, **sp. n.**, *P.marshalli* Schilthuizen & Perreau, **sp. n.**, *P.hanskii* Schilthuizen & Perreau, **sp. n.**, *P.sarawacensis* Schilthuizen & Perreau, **sp. n.**, *P.layangensis* Schilthuizen & Perreau, **sp. n.**, *P.microphallus* Schilthuizen & Perreau, **sp. n.**, and *P.alabensis* Schilthuizen & Perreau, **sp. n.** It is expected that the cholevine biodiversity of Borneo is still far from completely known. Nonetheless, provisional identification keys to all species known so far are presented.

## Introduction

Borneo is, after New Guinea, the second-largest tropical island of the world. It has never been strongly isolated, having formed part of a larger land mass, known as Sundaland, during marine transgressions in the Pleistocene (Hall and Holloway 1998). Sundaland comprises the present-day Malay Peninsula, the islands of Sumatra, Java, and Borneo, as well as the shallow seas in between. Recent paleoclimatic modelling (Raes et al. 2014) suggests that even during cooler periods of marine regression, evergreen wet forests dominated the area that now comprises Borneo. This, and the fact that the island, because of its mountainous character (with Gunung Kinabalu reaching almost 4,100 m) contains a great variety of habitats, has generated and maintained a rich biodiversity, not least in its soil and litter-dwelling invertebrate fauna (e.g., Hanski and Hammond 1986, Rahman et al. 2002, Liew et al. 2010).

The beetle subfamily Cholevinae (Coleoptera, Staphylinoidea, Leiodidae) consists mostly of small, soil-dwelling scavengers, well represented in the litter fauna of all tropical regions. Szymczakowski (1964) provided the first overview of the Cholevinae of South Asia, but the large number of new species described since then (Perreau 2000, and unpublished checklists) mean that this work is severely outdated by now. In recent years, several semi-comprehensive studies have appeared, either limited to a certain region (e.g., Wang and Zhou 2015) or to a certain habitat (e.g., Perreau 2009).

In this paper, we provide an overview of the species of Cholevinae currently known from the island of Borneo. By necessity, this is a very preliminary overview, since it is based on comparatively little information. Jeannel (1936) mentions only one species from Borneo. Szymczakowski (1961) mentions one more, and Peck (1981) and Perreau (2000) describe two more. In 2008, we (Schilthuizen and Perreau 2008) described seven new species and two new records from Borneo, bringing the total cholevine fauna to 13 species. However, more recent work (e.g., Merckx et al. 2015), as well as study of existing material in the Natural History Museum (London), deriving from the 1978 Mulu expedition (Hanski 1983, Hanski and Hammond 1986), and in Naturalis Biodiversity Center (Leiden), have revealed many additional new species. In our opinion, the 30 species that we recognize in the present paper form a sufficient basis to produce a first overview of our current, but doubtlessly still very incomplete, knowledge of the cholevine fauna of Borneo.

We provide a brief description for all previously described genera and species, and more extensive descriptions for newly-described species, as well as differential diagnoses for new species that have close congeners in Borneo. Where available, we also refer to DNA sequences in the Barcode of Life Database (BOLD, http://boldystems.org) and to so-called Barcode Index Numbers (BINs; Ratnasingham and Hebert 2013). We also give preliminary identification keys. However, given the fact that this overview is probably still far from complete, these keys should be used with caution: any sample collected in Borneo is likely to contain previously unrecognised species, and we hope this paper will stimulate further taxonomic and faunistic work.

The collection abbreviations used in the lists of examined material are as follows:

**BORN**Borneensis Collection, Institute for Tropical Biology and Conservation, Universiti Malaysia Sabah, Kota Kinabalu, Malaysia;

**FRCS**Forest Research Centre, Sepilok, Malaysia;

**HNHM**Hungarian Natural History Museum, Budapest, Hungary;

**JRUC** collection of Jan Růžička, Prague, Czech Republic;

**MHNG**Muséum d’Histoire Naturelle de Genève, Switzerland;

**NHMUK**Natural History Museum, London, UK (formerly British Museum (Natural History));

**RMNH**Naturalis Biodiversity Center, Leiden, The Netherlands (comprising the collections of the Rijksmuseum van Natuurlijke Historie, Leiden and the Zoölogisch Museum, Amsterdam).

**TXEX** Taxon Expeditions, Leiden, The Netherlands.

**CMPR** Collection Michel Perreau, Paris.

Male genitalia were mounted in Euparal after dissection and dehydration in ethanol 95%. Female genitalia were cleared in hot KOH 10% and stained with Azoblack before mounting in DMHF (2,5-DiHydroxyMethylFurane). Photographs of genitalia were taken on a Leitz Diaplan microscope using a Spot Insight IN1820 or a Leica MC170HD camera. High-resolution photonic pictures of external morphological details (Figure 14a–e) were taken with a Keyence VHX5000 microscope and a VH-Z250T lens. The outline of the map was built from SRTM3 data (Shuttle Radar Topography Mission) of NASA with the software QGIS. They were completed by the global administrative areas database GDAM (www.gdam.org) and the hydrographic networks of Natural Earth (http://www.naturalearthdata.com/). All images and descriptions will be uploaded to the Cholevidae of the World website (http://cholevidae.myspecies.info/).

## Systematics

### Tribe: ANEMADINI Hatch

#### Genus: Micronemadus

Taxon classificationAnimaliaColeopteraLeiodidae

Jeannel, 1936

##### Description.

Small, 1.4–2.6 mm. Oval habitus, with the apex of the elytra rounded. Head and pronotum punctate, elytra with transverse strigae. Antenna with antennomeres 4 and 5 very short and wide. Mesosternum with a longitudinal mesoventral process. The four first protarsomeres and the first mesotarsomere dilated in the male. Aedeagus: median lobe triangular, parameres longer than the median lobe, and bent inward.

#### Micronemadus sondaicus

Taxon classificationAnimaliaColeopteraLeiodidae

Schilthuizen & Perreau sp. n.

http://zoobank.org/8AA2A703-76B4-4DCE-BFD3-8E19943EA8BA

Figure 1a, c–f

##### Material.

**Holotype**: Malaysia, Sabah, Crocker Range Park, Inobong, 5°51.265'N, 116°08.363'E, 500 m elev., 21–23.ix.2012 (leg. M. Schilthuizen, Crocker Range / Kinabalu Expedition, RMNH.INS.555641), male. **Paratypes**: *Sabah.* Crocker Range Park, Inobong, 5°51.3'N, 116°08.4'E, 500 m elev., 21–23.ix.2012 (leg. M. Schilthuizen, Crocker Range / Kinabalu Expedition, RMNH.INS.555640, 555642), 2 individuals; Crocker Range, Gunung Alab, xii.2009 (leg. M. Schilthuizen, RMNH.INS.63291, 63310, 63295), 3 individuals; Crocker Range, along the road from Kota Kinabalu to Tambunan, near Rafflesia Park, 5°46.4'N, 116°20.8'E, 1350 m elev., baited pitfall trap, 2001 (leg. M. Schilthuizen, RMNH.INS.549293–549295), 3 individuals; Kinabalu Park, Headquarters Area, Liwagu Trail, 6°0.487'N, 116°32.7'E, pitfall with chicken, 2.iv.2016 (leg. M. Schilthuizen, I. Njunjić & F. Feijen, BORN, RMNH.INS.1086143–1086158), 36 individuals. Kinabalu Park, Sayap, ix.2012 (leg. M. Schilthuizen, Crocker Range / Kinabalu Expedition, BOR/COL/14178–14193), 16 individuals. *Sarawak.* Gunung Mulu National Park, many localities between 100 and 950 m elev., iii–viii.1978 (leg. P. M. Hammond & J. E. Marshall, NHMUK BM 1978–49), 131 specimens.

##### Description.

**Figure 1.**
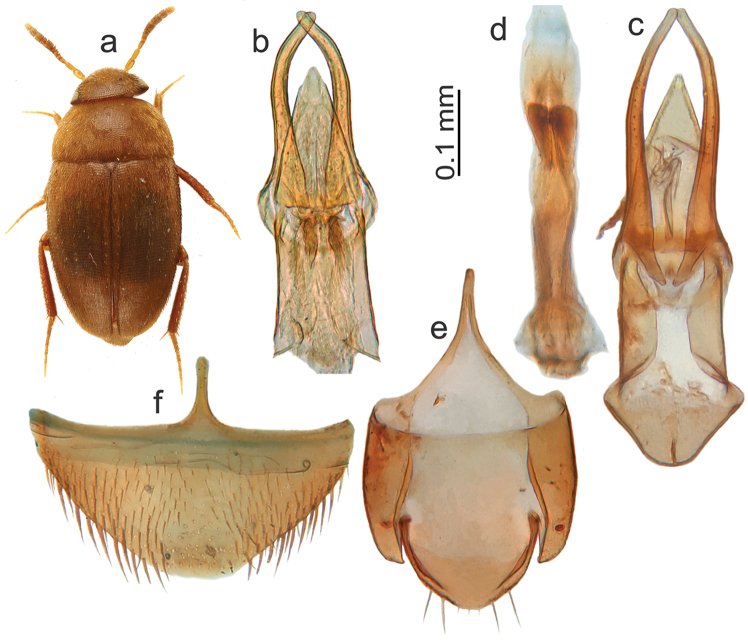
*Micronemadus*. **a***M.sondaicus* sp. n. habitus (paratype female, Sarawak, Gunung Mulu) **b***M.pusillimus* (Kraatz) aedeagus, Sarawak, Gunung Mulu **c***M.sondaicus* sp. n. aedeagus Sarawak, Gunung Mulu **d***M.sondaicus* sp. n. endophallus, Sarawak, Gunung Mulu **e***M.sondaicus* sp. n. male urite IX, Sarawak, Gunung Mulu **f***M.sondaicus* sp. n. female sternite VIII, Sarawak, Gunung Mulu.

Length: 2.0–2.6 mm. Colouration: in fully coloured individuals dark brown to black, margins of pronotum and front half of the elytra reddish brown, entirely covered in reddish brown setae; legs, palps, and basal three and final antennomeres light reddish brown; antennomeres 4–10 darker (Figure 1a). Pronotum: small, much narrower than the elytra, ca. 2 times as broad as wide, with the greatest width slightly frontal of the caudal angles; basal margin gently emarginated near the angle, and with an angular sinuosity in the centre, on either side of the scutellum. Elytra: rounded at the apex, 1.25 times as long as jointly wide (length measured from the caudal tip of the scutellum to the apex of the elytra). Winged. Male protarsi: slightly narrower than the protibia, segments 1–4 as long as wide. Aedeagus: median lobe pointed, nearly perfectly triangular, parameres slender, extending far beyond the tip of the median lobe (Figure 1c). Female sternite VIII caudally broadly rounded, rostrally tapering into a narrow tip.

##### Differential diagnosis.

Similar to *M.pusillimus* (Kraatz, 1877), described from Japan, but compared with Japanese specimens of *M.pusillimus* that we have seen, *M.sondaicus* is larger, more uniformly coloured (Figure 1a; Japanese *M.pusillimus* have a more strikingly dark pronotum), has a simple triangular shape of the median lobe of the aedeagus (in *M.pusillimus*, the apex is separately acuminate; see Figure 1b), and more slender parameres (Figure 1c). The female sternite VIII in *M.pusillimus* is more slender and rostrally not as narrowly tapered as in *M.sondaicus*. *M.ruzickai* Perreau, 2004 differs by the parameres that are even more massive than in *M.pusillimus*.

##### DNA-barcodes.

In the BOLD database, COI sequences are available for the holotype (RMNH.INS.555641) and two paratypes (RMNH.INS.555640, 555642) from Inobong (Crocker Range) and also for the paratypes RMNH.INS.549293–549295 from Gunung Alab (Crocker Range). See also under Remarks.

##### Habitat and distribution.

Very common and widespread, in primary and secondary forest, 0–1850 m elev. In addition to the type material, we have seen seemingly conspecific material from many other localities in Sundaland, (Sabah, Sarawak, Peninsular Malaysia, Mindanao, Java, Bali) and also from Vietnam. Nishikawa’s (1989) records of *M.pusillimus* from Peninsular Malaysia, Sabah, Sarawak, Java, and Bali (which we did not see) may also refer to *M.sondaicus*. This overview suggests that *M.sondaicus* is widespread in Southeast Asia (but see below under remarks).

##### Remarks.

For many years, we considered this Borneo *Micronemadus*, which is usually the commonest leiodid in baited traps, as identical to *M.pusillimus*. However, DNA-barcodes for Japanese individuals (BOLD BIN ABU9390) and Bornean individuals (BOLDBINsABU9391 and ACK0008) display a 17% sequence divergence, strongly suggesting that, despite the only slight morphological differences, these belong to separate, not closely related species. We suspect that *M.pusillimus*, previously considered a very widespread Asian species (Szymczakowski 1964), may represent a complex of genetically strongly differentiated, but morphologically very similar taxa. In fact, among the DNA-barcoded specimens of *M.sondaicus* from Sabah’s Crocker Range, we already see a 2.7% sequence divergence between highland and lowland populations, which has led the BOLD algorithm to place them into separate BINs (BOLD:ABU9391 and ACK008, respectively). At the moment, however, we consider ABU9391 and ACK008 as conspecific.

##### Etymology.

The name refers to the Sunda region, of which Borneo forms part (*sondaicus* (L.) = from Sunda). We used the spelling *sondaicus*, rather than *sundaicus*, to conform with other specific epithetons, such as *Rhinocerossondaicus* Desmarest, 1822.

### Tribe: CHOLEVINI Kirby, 1837

#### Genus: Catops

Taxon classificationAnimaliaColeopteraLeiodidae

Paykull, 1798

##### Description.

Fairly robust cholevines of sizes that range from just longer than 2 mm to almost 10 mm. Body fairly convex, pronotum relatively large, but usually narrower than the elytra. Elytra sometimes with traces of parallel longitudinal striae. Antennae with the 8^th^ antennomere usually broader than long. In the male, the four first protarsomeres and the first mesotarsomere are dilated; also, the protibiae often are sexually dimorphic. Aedeagus: median lobe usually symmetric, elongated, lance-shaped, terminally rounded, truncate, acute, or two-pronged. Parameres usually thin, hair-like.

#### Catops pruinosus

Taxon classificationAnimaliaColeopteraLeiodidae

Schweiger, 1956

Figure 2

Catops pruinosus Schweiger, 1956: 538, fig. 5; type from Kuatun, Fukien, China.
Catops solitarius Szymczakowski, 1961: 129, figs 16–19; type from Sandakan, Sabah, Borneo (in NHMUK).

##### Material.

*Sabah.* Sandakan (leg. W.B. Pryer, NHMUK 1925–264). Holotype of *C.solitarius* Szymczakowski (examination based on a photograph taken by Jan Růžička).

##### Description.

Length: 3.9 mm. Habitus slender and elongated, somewhat flattened. Body reddish brown; head, centre of the pronotum, and centre of the elytra dark brown. Entire dorsum covered in orange setae. Antenna robust, 7^th^ antennomere as wide as long, 6^th^, 8^th^, 9^th^, and 10^th^ wider than long. Pronotum 1.54 times as wide as long, frontal and caudal margins straight, lateral margins curved, more strongly rostrad than caudad (greatest width of the pronotum slightly behind the centre), caudal angles broadly rounded; surface with dense, rasp-like punctuation and somewhat matte due to microsculpture. Elytra with fine punctuation, shagreened, and with slight indications of longitudinal striae, 1.27 times as wide as the pronotum, 1.3 times as long as jointly wide (length measured from the caudal tip of the scutellum to the apex of the elytra). Male unknown.

##### Habitat and distribution.

*Catopspruinosus* is known from a large latitudinal expanse along the East Asian coast: Shanghai, Fujian, and North Borneo. The only known record from Borneo (Sabah: Sandakan) is the female holotype of *C.solitarius*, later synonymized with *C.pruinosus* (Szymczakowski 1964).

##### Remarks.

**Figure 2.**
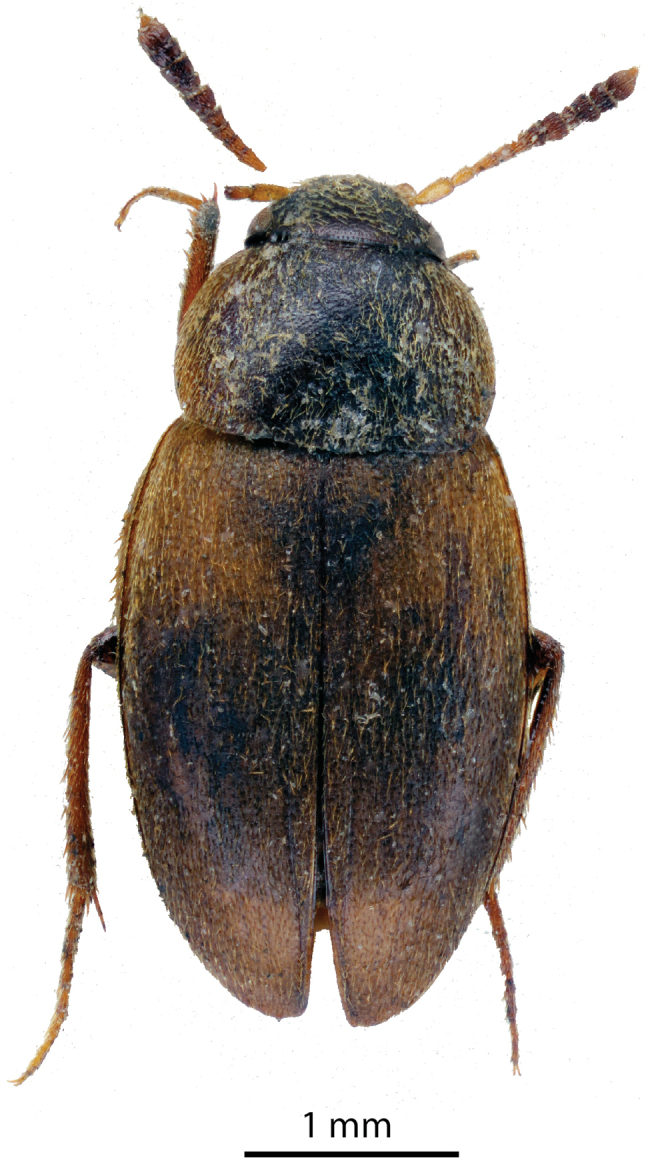
*Catopspruinosus* Schweiger, habitus of the female holotype of *C.solitarius* Szymczakowski from Sandakan (photograph Jan Růžička).

*Catopspruinosus* is a member of the *C.hilleri* group (Szymczakowski 1964), represented by ca. 30 species, primarily from central and eastern Asia (Perreau 2000). The diagnosis above is based on a photograph (Figure 2) of the holotype of *C.solitarius*, as well as the description of *C.solitarius* by Szymczakowksi (1961). In the absence of males, we are unsure whether the Borneo specimen is indeed conspecific with *C.pruinosus*. Indeed, we also doubt that the specimen truly derives from Borneo: despite many years of work in Borneo, we have never come across any Cholevini. If members of such temperate-region groups exist in Borneo, we would expect them to occur in the highlands, rather than in coastal locations such as Sandakan. In fact, it is not impossible that the Sandakan specimen is a mislabelled Chinese specimen, as the collector, William Burgess Pryer, was active in Shanghai (where *C.pruinosus* is known to occur) immediately before moving to Sandakan (Tregonning 1954).

### Tribe: PTOMAPHAGINI Jeannel, 1911

#### Genus: Baryodirus

Taxon classificationAnimaliaColeopteraLeiodidae

Perreau, 2000

##### Description.

**Figure 3.**
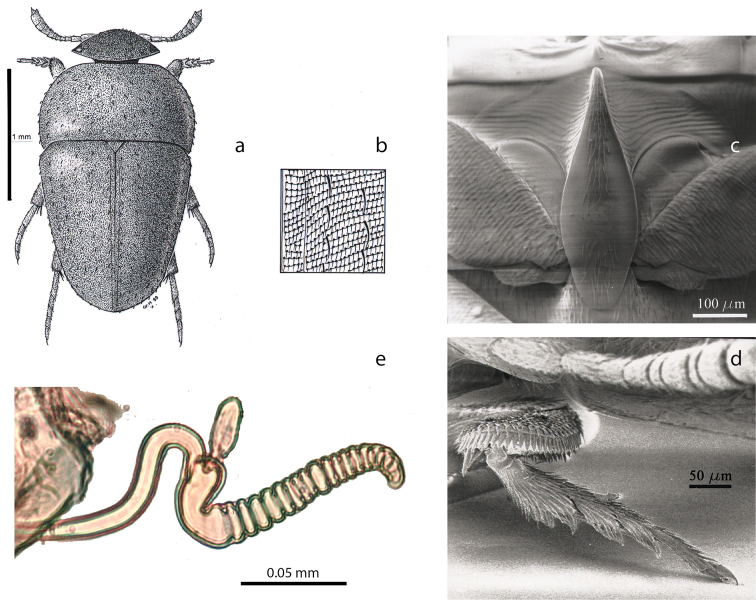
*Baryodirushammondi* Perreau (holotype female, Sarawak, Gunung Mulu; after Perreau 2000). **a** habitus (drawing by G. Hodebert) **b** detail of double setation on elytra **c** mesoventral process, ventral view **d** protarsus **e** spermatheca.

Length: 2.2 mm. Winged and with fully developed eyes (Figure 3a). Body uniformly brown, eyes black. Dorsal surface with transverse strigae. Pronotal and elytral surface with two types of setation: one dense, long, and recumbent aligned on the transverse strigae, and one sparse, long and erected, roughly aligned along longitudinal rows (Figure 3b). Head with a high and robust occipital carina. Pronotum convex and transverse, slightly wider than the elytra at the shoulders, 1.9 times as wide as long, the lateral sides rounded, the largest width near the anterior third of the length. Basal margin without a marginal gutter, slightly sinuate near the slightly drawn-out lateral angles. Elytra exactly as long as wide, the largest width at the base. Elytral sides nearly straight, weakly arcuate, giving the elytra a triangular shape. The sutural stria is the only recognizable elytral stria. Mesoventral process strikingly wide and high, anteriorly angular, with flat and setose ventral side, posteriorly widely expanded above the metasternum (Figure 3c). Protibiae with a lateral row of spines along the external edge and with smaller spines randomly arranged on the ventral side (Figure 3d). Mesotibiae and metatibiae with a circular row of spines around the apex. Female protarsi tetramerous and strikingly expanded with dense setae on the ventral side. Male unknown. Spermatheca elongated with a succession of rings along the entire length (Figure 3e; a somewhat similar condition is found in *Ptomaphaminuslatescens* Szymczakowski, 1964 and *P.testaceus* sp. n.).

#### Baryodirus hammondi

Taxon classificationAnimaliaColeopteraLeiodidae

Perreau, 2000

Figure 3a–e

Baryodirus hammondi Perreau, 2000 19–20, figs 1–10; type from Mulu, Sarawak (in NHMUK).

##### Description.

See genus description above: the genus is monotypic, and the holotype is the only known specimen of the species.

##### Habitat and distribution.

The biotope and ecology of this species are unknown, but several characters suggest that it could be a commensal of hymenopterans. The compact habitus and the double setation are observed (almost exclusively) in many myrmecophilous leiodid genera or subgenera, such as Ptomaphagus (Echinocoleus) Horn in North America, and *Synaulus* in North Africa (our observations).

#### Genus: Ptomaphaginus

Taxon classificationAnimaliaColeopteraLeiodidae

Portevin, 1914

##### Description.

In Borneo, *Ptomaphaginus* consists of relatively large Ptomaphagini with four dilated male protarsomeres, distinguishable from *Ptomaphaminus* by the ventral spines of the protibiae being aligned along the latero-external row of equal spines, making a second, more widely spaced row next to the external one, as well as by the metaventral sutures, which are roughly parallel to the axis of the body. Also, in the female, the gonocoxites are elongated, whereas in most *Ptomaphaminus*, they are reduced.

With *Ptomaphaminus* Perreau (see below), *Ptomaphaginus* Portevin makes up the vast majority of the Borneo cholevine diversity. We currently recognise 14 species. However, more diversity is to be expected, both cryptic (e.g., DNA-analysis suggests additional diversity in *P.bryanti* and related species; Merckx et al. 2015) and non-cryptic (each field project in previously unexplored regions in Borneo results in new discoveries).

#### Ptomaphaginus anas

Taxon classificationAnimaliaColeopteraLeiodidae

Schilthuizen & Perreau, 2008

**Figure 4.**
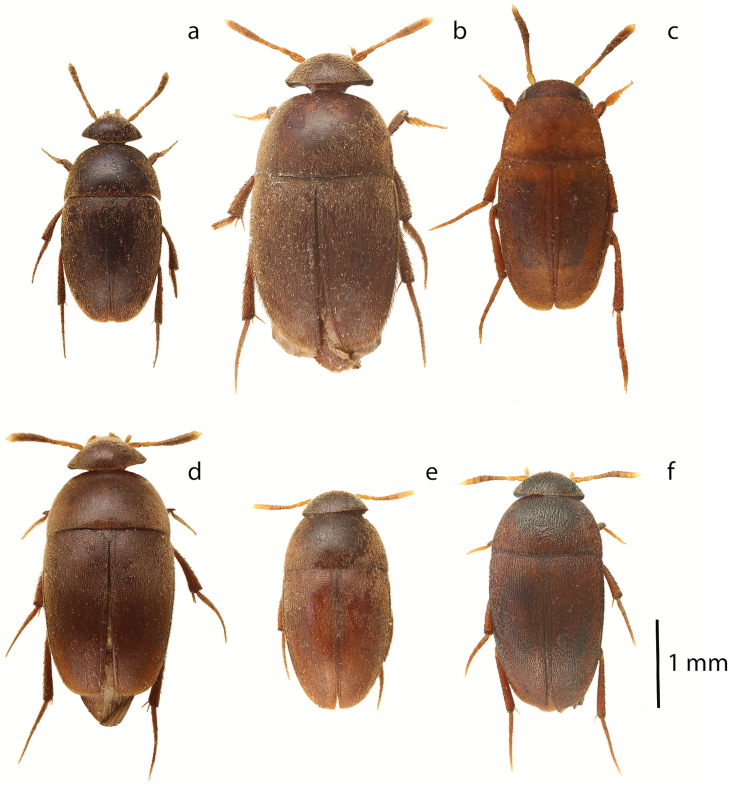
*Ptomaphaginus*, habitus, dorsal. **a***P.anas* Schilthuizen & Perreau, Ulu Gombak, male (RMNH) **b***P.bryantioides* Schilthuizen & Perreau, Gunung Mulu, male (NHMUK) **c***P.caroli* Schilthuizen & Perreau, Gunung Mas, holotype, male (RMNH) **d***P.grandis* sp. n., Gunung Mulu, paratype, male (NHMUK) **e***P.kinabaluensis* Schilthuizen & Perreau, Gunung Kinabalu, paratype, male (RMNH) **f***P.kinabaluensis* Schilthuizen & Perreau, Gunung Kinabalu, paratype, female (RMNH.INS.1086205).

Figures 4a , 6i–j , 9a

Ptomaphaginus anas Schilthuizen & Perreau, 2008: 199, figs 16–17; type from Gombak, Selangor, Peninsular Malaysia (in RMNH).

##### Material.

(In addition to that given in Schilthuizen and Perreau (2008)): *Sarawak*. Kuching, Semongok, 1°24.7'N, 110°19.3'E, 16.i.1978, (RMNH.INS.549311) 1 male.

##### Description.

(Adapted from Schilthuizen and Perreau (2008)). Medium-sized (2.3–2.9 mm) and dark-coloured species. Pronotum 1.65 times as wide as long, as wide as the elytra. Elytra 1.1 times as wide as their combined length (length measured from the caudal tip of the scutellum to the apex of the elytra). Winged. Aedeagus in dorsal view gradually narrowing towards the apex. The tip is triangular and subtly asymmetric. A broad medial furrow runs over the dorsal side of the aedeagus, and dissolves just short of the apex. In lateral view, the median lobe is strongly curved, almost semicircular, and distinctly thickened at the point of strongest curvature.

##### Differential diagnosis.

The aedeagus is characteristically shaped: strongly curved, distally tapering into a narrow, flattened, and slightly upturned apex. *Ptomaphaginuslouis* and *P.muluensis* have a similar aedeagus, which, however, is less strongly curved in the terminal half.

##### DNA barcodes.

One individual (RMNH.INS.549311; see under Examined material) is available in BOLD, but no DNA has been successfully sequenced from this specimen.

##### Habitat and distribution.

Lowland and lower montane forest, up to 1,500 m. Sabah: Batu Punggul, Kinabalu Park HQ, Kibongol; Sarawak: Semongok. Peninsular Malaysia: Gombak, Cameron Highlands.

#### Ptomaphaginus bryanti

Taxon classificationAnimaliaColeopteraLeiodidae

Jeannel, 1936

Ptomaphaginus Bryanti Jeannel, 1936: 56–59, figs 63–66; type from Mt. Matang, Sarawak, Borneo (in NHMUK).

##### Description.

Length 2.8 mm. Winged. Slender build (pronotum 1.56 times wider than long), as wide as the elytra. Aedeagus subterminally with two lateral extensions and a long median processus (the image given by Jeannel [1936] is erroneous, due to mounting error [Schilthuizen and Perreau 2008]).

##### Differential diagnosis.

Very similar to *P.bryantioides*, from which it differs chiefly (as far as can be discerned from the single *P.bryanti* individual available) by its more slender habitus.

##### DNA barcodes.

In the BOLD database, COI barcodes are available for RMNH.INS.555591 and 555594, two specimens that possibly belong to this species (See under Habitat and distribution).

##### Habitat and distribution.

To date only known from the type location, Mt. Matang in Sarawak. However, given the great molecular distances (Merckx et al. 2015) that we find among Sabah populations of *P.bryantioides*, a more robust species with an aedeagus nearly indistinguishable from *P.bryanti* (see below), we suspect that some specimens assigned to that species, especially those from Sugud (BOLD BIN ACJ9515, RMNH.INS.555591 and 555594), may in fact belong to *P.bryanti* (although they are not as slender as the holotype of *P.bryanti*).

#### Ptomaphaginus bryantioides

Taxon classificationAnimaliaColeopteraLeiodidae

Schilthuizen & Perreau, 2008

Figures 4b , 6c–d , 9b , 10a

Ptomaphaginus bryantioides Schilthuizen & Perreau, 2008: 192–193, figs 20–21; type from Danum Valley, Sabah, Borneo (in RMNH).

##### Material.

(In addition to that given in Schilthuizen and Perreau (2008)): *Sabah.* Sandakan, Sungai Lokam (logged forest), flight interception trap, iii.1997 (leg. A.Y.C. Chung, FRCS), 1 male; Sandakan, Sepilok (primary forest), flight interception trap, x.1996 (leg. A.Y.C. Chung, FRCS), 1 female. Kinabalu Park, Poring Hot Springs, 6°02.894'N, 116°41.957'E, 625 m elev., in baited pitfall traps, 15–20.ix.2012, (leg. M. Schilthuizen, Crocker Range / Kinabalu Expedition, RMNH.INS.555625–555628), 4 individuals; Crocker Range Park, Inobong, 5°51.265'N, 116°08.363'E, 500 m elev., 21–23.ix.2012 (leg. M. Schilthuizen, Crocker Range / Kinabalu Expedition, RMNH.INS.555637), 1 individual; Kiansom Waterfall, 5°58.444'N, 116°12.526'E, 300 m elev., 5–7.ix.2012 (leg. M. Schilthuizen, Crocker Range / Kinabalu Expedition, RMNH.INS.555598–555600), 3 individuals; Kiansom Waterfall, 5°58.444'N, 116°12.526'E, 300 m elev., 21–28.xii.2009 (leg. M. Schilthuizen, RMNH.INS.555644–555645, 549278, 63309, 63289–63290), 6 individuals; Crocker Range, Kota Kinabalu–Tambunan road (km 56), 1350 m elev., fish and human excrement traps, 21–24.xi.1987 (leg. Krikken & Rombaut, RMNH.INS.1086159), 1 male; Kota Kinabalu, Tun Fuad Stephen Park, 20.iv.2003 (leg. Ng Kok Kit, RMNH.INS.63303, 63298, 549268–549271), 6 individuals; Kota Kinabalu, Tun Fuad Stephen Park, 5°56.717'N, 116°06.709'E, 75 m elev., baited pitfall traps, 23–29.xii.2009 (leg. M. Schilthuizen, RMNH.INS.555646–555677, 63285–63286, BOR/COL/14194–14205), 46 individuals. *Sarawak*. 4^th^ Division, Gunung Mulu National Park, many localities between 100 and 800 m elev., iii–viii.1978 (leg. P. M. Hammond & J. E. Marshall, NHMUK BM 1978–49), 102 males, 116 females. *Kalimantan Timur*. Balikpapan env., ca. 25 km by road Sungain Wain reserve, camp Djamaludin, baited traps in primary dipterocarp forest, a clearing next to small stream, 14–17.ii.2010 (leg. P. Šipek, H. Šipkovà, JRUC), 1 male, 2 females.

##### Description.

Habitus broad, rectangular, flat, very variable in size, 2.1–3.5 mm. Pronotum on average 1.8 times as wide as long, as wide as the elytra. Elytra as long as wide (length measured from the caudal tip of the scutellum to the apex of the elytra). Winged. Aedeagus with two apical, laterally directed extensions and a long terminal processus. (It appears that in *P.bryanti*, the lateral extensions may be directed even more ventrad, and the median processus is more widened apically than in *P.bryantioides*, but the significance of these differences needs to be substantiated.) Male forelegs with long setae on the ventral sides of the femur and tibia. The 3^rd^, 4^th^, and 5^th^ visible abdominal ventrite of the male carry a slight central notch and show a depression around these notches. Spermatheca simple, inflated, semicircular (Figure 10a), similar to that of *P.louis*.

##### Differential diagnosis.

Distinguishable from other Bornean *Ptomaphaginus* species with the same aedeagus structure by the more elongated aedeagus with long, narrow processus, and the long setae on the male femur and tibia.

##### DNA barcodes.

For the following specimens, COI barcodes are available in BOLD: RMNH.INS.549278 (Kiansom), RMNH.INS.63289 (Kiansom), RMNH.INS.555644–555645 (Kiansom), RMNH.INS.555598–555600 (Kiansom), RMNH.INS.63285–63286 (Kota Kinabalu), RMNH.INS.549268–549271 (Kota Kinabalu), RMNH.INS.555677 (Kota Kinabalu), RMNH.INS.555625–555628 (Poring Hot Springs), and RMNH.INS.555637 (Inobong). Although these sequences fall into several separate BINs, for the moment we consider them all conspecific (see under Habitat and distribution).

##### Habitat and distribution.

Widely distributed in Sabah and northern Sarawak, in primary and secondary lowland forest (usually up to 500 m, one exceptional record at 1350 m). Sabah: Kota Kinabalu, Kiansom, Sugud, Kinabalu Park (Poring), Crocker Range Park (Inobong, KK–Tambunan road km 56), Danum Valley, Batu Punggul; Sarawak: Mulu National Park. DNA sequencing (Merckx et al. 2015) shows that within Sabah, several lineages exist. The most widely divergent of these are from Sugud and may represent a separate species (possibly *P.bryanti*; see above). Additionally, an approximate 4% COI-distance can be observed between the populations on the east and west slopes of the Crocker Range (BOLDBINsACJ9516+ACJ9517 and ABU8889+ABU8890, respectively), but morphologically these populations are indistinguishable. For the time being, we consider all these BINs as conspecific.

#### Ptomaphaginus burckhardti

Taxon classificationAnimaliaColeopteraLeiodidae

Schilthuizen & Perreau, 2008

**Figure 5.**
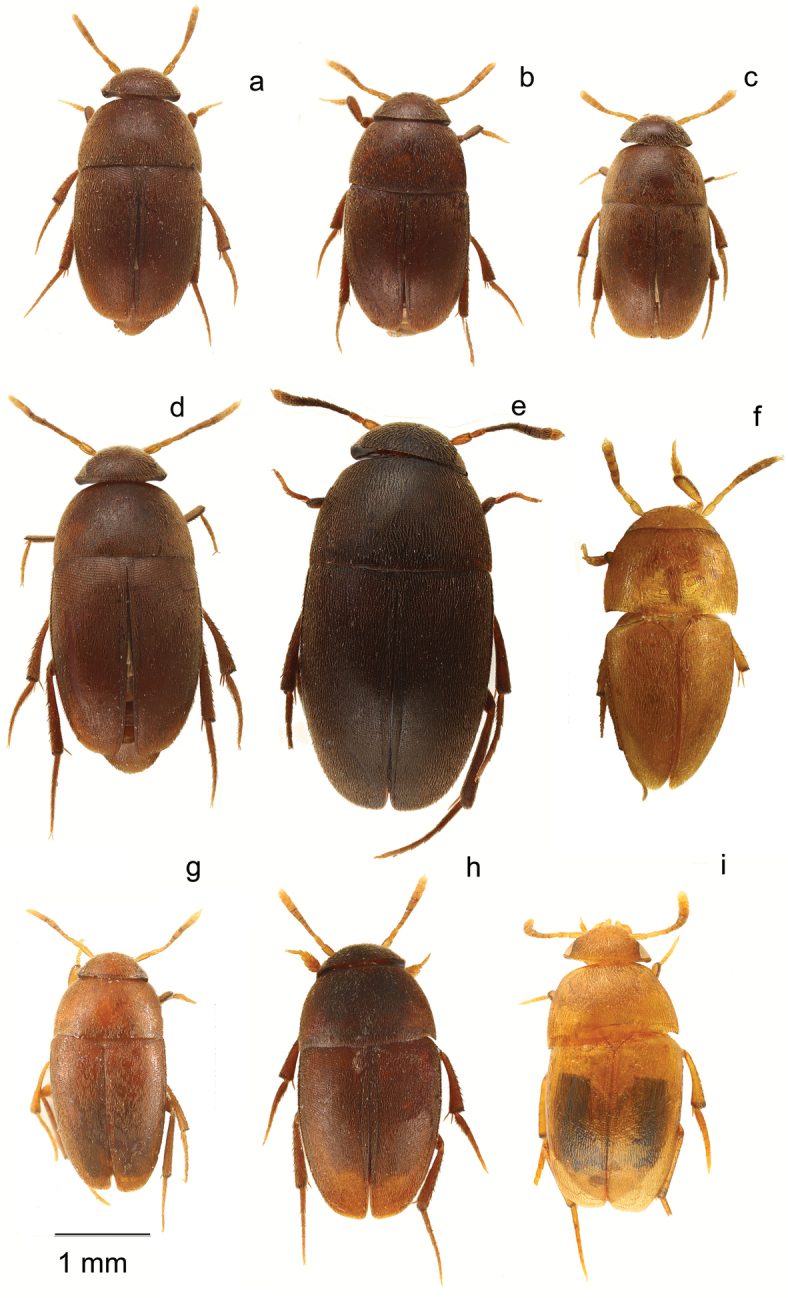
*Ptomaphaginus*, habitus, dorsal. **a***P.louis* sp. n., Gunung Mulu, paratype, male (NHMUK) **b***P.muluensis* sp. n., Gunung Mulu, paratype, male (NHMUK) **c***P.scaphaner* Szymczakowski, Gunung Mulu, male (NHMUK) **d***P.similipes* Schilthuizen & Perreau, Gunung Mulu, male (NHMUK) **e***P.isabellarossellini* sp. n., Gunung Kinabalu, paratype, female (RMNH.INS.1086164) **f***P.burckhardti* Schilthuizen & Perreau, Gunung Kinabalu, male, holotype (MNHG) **g***P.sabahensis* Schilthuizen & Perreau, Gunung Kinabalu, male, holotype (MNHG) **h***P.latimanus* Schilthuizen & Perreau, Gunung Trus Madi, holotype, male (RMNH) **i***P.latimanus* Schilthuizen & Perreau, Gunung Trus Madi, paratype, female (RMNH.INS.1086206).

Figures 5f , 7g, h , 9c

Ptomaphaginus burckhardti Schilthuizen & Perreau, 2008: 202–203, figs 7–8; type from Gunung Kinabalu, Sabah, Borneo (in MHNG).

##### Description.

(Adapted from Schilthuizen and Perreau (2008)). Length 2.5 mm. Habitus slender, ovoid. Pronotum 1.7 times as wide as long, slightly broader than the elytra. Elytra slender, 1.25 times as long as their combined width (length measured from the caudal tip of the scutellum to the apex of the elytra). Uniformly light brown. Wingless. Eyes reduced. Elytra laterally not curved, narrowed caudad in an approximately straight line. Male with a large and deep semicircular depression extended on the 5^th^ and 6^th^ visible abdominal sternites, bordered on the front half with long and dense setae, and a central notch on the apical edge of the 6^th^. Aedeagus slender. The apex is tapered terminally and ends in a flattened, duck-bill-shaped processus. It carries several long, curved, lateral setae.

##### Differential diagnosis.

Unique among the Bornean *Ptomaphaginus* because of its small, slender build, reduced eyes, and long, slender aedeagus.

##### Habitat and distribution.

Only two specimens known (holotype and paratype), from upper montane forest at 2600 m elev. on Gunung Kinabalu.

#### Ptomaphaginus caroli

Taxon classificationAnimaliaColeopteraLeiodidae

Schilthuizen & Perreau, 2008

Figures 4c , 8a, b

Ptomaphaginus caroli Schilthuizen & Perreau, 2008: 193, figs 14–15, 26; type from Gunung Mas, Sabah, Borneo (in RMNH).

##### Description.

(Adapted from Schilthuizen and Perreau (2008)). Length 2.7 mm. Habitus relatively slender and narrow, flat. Pronotum 1.62 times as long as wide, slightly narrower than the elytra. Elytra 1.41 times as long as their combined width (length measured from the caudal tip of the scutellum to the apex of the elytra). Winged. Long setae on the ventral side of the male profemur and protibia absent. Aedeagus apically with two short ‘wings’ and a very small, indistinct terminal processus. *Spiculum gastrale* long-triangular, the apex nearly truncate, with a small central projection; similar in shape to *P.latimanus* and *P.similipes*.

##### Differential diagnosis.

*Ptomaphaginuscaroli* has a similar aedeagus as *P.bryanti*, *P.similipes*, and *P.bryantioides*. However, it differs in having a distinctly elongated habitus (elytral index of 1.41) and very short apical ‘wings’ on the aedeagus.

##### Habitat and distribution.

So far, only known from the type specimen, collected in lower montane forest at 1350 m in the Crocker Range of Sabah. The aedeagal shape shows that it belongs within the “*bryanti*-group”.

##### Remarks.

The aedeagus of the holotype has been lost shortly after it was first collected and studied (in 2000). Before the loss, sketches were made of the dorsal and lateral view of the aedeagus, which form the basis for the line drawing in Figure 8 and in Schilthuizen and Perreau (2008).

#### Ptomaphaginus grandis

Taxon classificationAnimaliaColeopteraLeiodidae

Schilthuizen & Perreau sp. n.

http://zoobank.org/1995F69B-A8D0-44E7-B4D5-A8BA57C2235C

**Figure 6.**
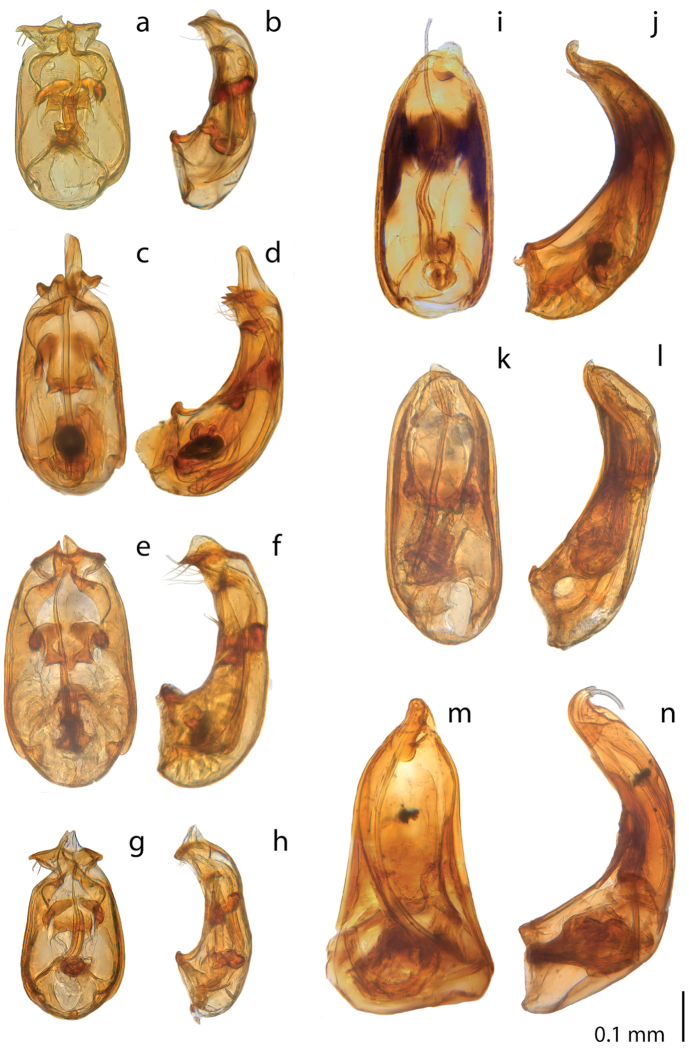
*Ptomaphaginus*, aedeagus, dorsal (left) and lateral (right) view. **a, b***P.kinabaluensis* Schilthuizen & Perreau, Gunung Kinabalu (RMNH.INS.63302) **c, d***P.bryantioides* Schilthuizen & Perreau **e, f***P.similipes* Schilthuizen & Perreau, Gunung Mulu (NHMUK) **g, h***P.latimanus* Schilthuizen & Perreau Gunung Trus Madi, holotype (RMNH) **i, j***P.anas* Schilthuizen & Perreau, Semongok (RMNH) **k, l***P.grandis* sp. n. Gunung Mulu, paratype (NHMUK) **m, n***P.louis* sp. n. Gunung Mulu, paratype (NHMUK).

Figures 4d , 6k, l , 9d , 10b

##### Material.

**Holotype**: Malaysia, Sarawak, Mulu National Park, Slope, TPS 7–9, 29.iv.1978 (P.M. Hammond & J.E. Marshall leg., NHMUK, B.M. 1978–49), male. **Paratypes**: Sarawak. Mulu National Park, Slope, TPS 7–9, 29.iv.1978 (leg. P.M. Hammond & J.E. Marshall, B.M. 1978–49, NHMUK), 5 males, 7 females; Mulu National Park, Slope, TPS 10–12, v–viii.1978 (leg. P.M. Hammond & J.E. Marshall, B.M. 1978–49, NHMUK), 1 male, 5 females.

##### Description.

Habitus: very large (3.4–4.0 mm), dark reddish brown, the elytra deeper red than the pronotum, basis and tip of the antenna pale, antennomeres 5–10 much darker; relatively parallel-sided and somewhat convex, head relatively narrow, pronotum 1.67 times as wide as long, slightly wider than the elytra, caudal angles clearly extended. Elytra of moderate length, convex, broadest at the shoulders, in the caudal one-third gently rounded towards the apex, jointly ca. 1.5 times as long as wide (length measured from the caudal tip of the scutellum to the apex of the elytra). Body entirely covered in dense pale yellow setation. Wings present. Antennae slender, 4^th^ antennomere longer than wide, 9^th^ and 10^th^ antennomere square. Male protarsi only slightly dilated, the first four tarsomeres jointly ca. 4 times as long as wide. Aedeagus short and broad, in lateral view only very slightly bent ventrad with a barely perceptible upturned tip at the end, in dorsal view narrowing (in rounded fashion) towards the blunt apex. Stylet short and straight. *Spiculum gastrale* long, narrow-triangular, with the caudal part rounded. Spermatheca U-shaped, with ca. 6 distinct narrow rings on the proximate leg of the “U”, and ca. 6 additional, indistinct, broader rings on the remainder. Spermiduct long, thin, consisting of 5–6 360° coils.

##### Differential diagnosis.

Externally distinctive by its large size and the colour pattern of the antennae. From equally large *P.bryantioides*, it may be distinguished by the narrower male protarsi and the longer elytra. Aedeagus cannot be confused with that of any other known Bornean species, but is similar in shape to the one of *P.nitens* Jeannel, 1936 from Sri Lanka (which, however, is smaller and has much more condensed antennae) and of several species described from China and Taiwan (especially *P.pingtungensis* Perreau, 1996, *P.guangxiensis* Wang & Zhou, 2015, *P.perreaui* Wang & Zhou, 2015, and *P.yui* Wang & Zhou, 2015), from which *P.grandis* is distinguished by the unique combination of body size, antennomere proportions, and details of the *spiculum gastrale*, spermatheca, and aedeagus shape.

##### Habitat and distribution.

Only known from Mulu National Park in Sarawak.

##### Etymology.

Named *grandis* for its large size.

#### Ptomaphaginus isabellarossellini

Taxon classificationAnimaliaColeopteraLeiodidae

Schilthuizen, Njunjić & Perreau sp. n.

http://zoobank.org/87C3F9D7-6CBD-4351-B96F-307A65F0716D

**Figure 7.**
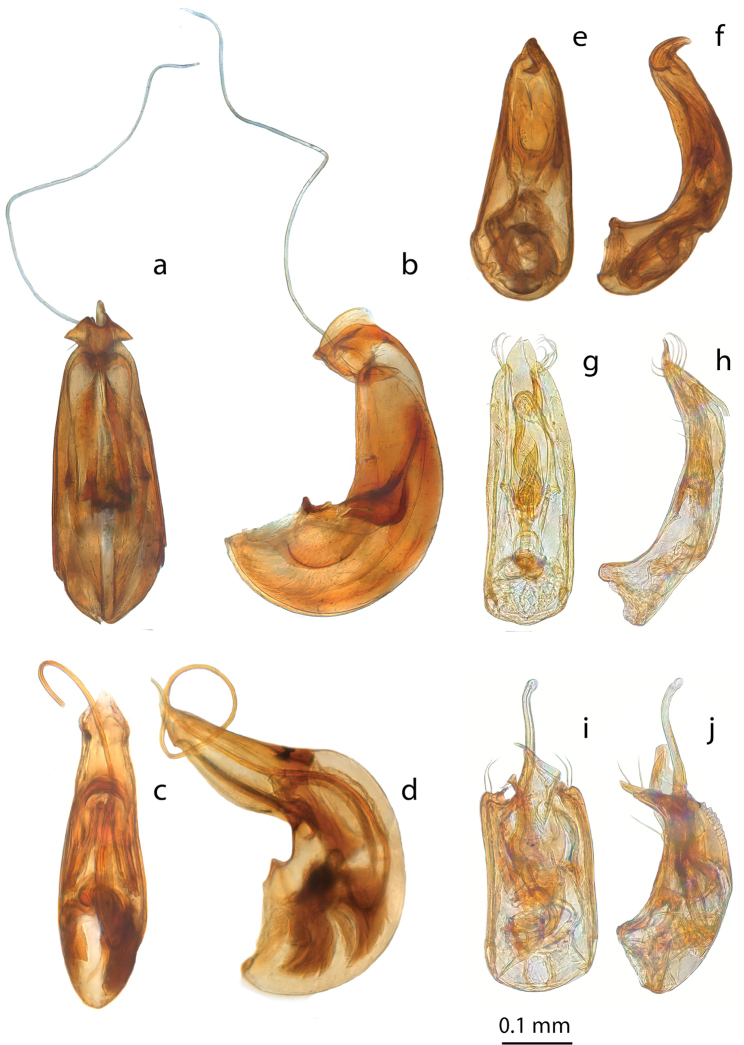
*Ptomaphaginus*, aedeagus, dorsal (left) and lateral (right) view. **a, b***P.isabellarossellini* sp. n. Gunung Kinabalu, holotype (RMNH.INS.1086160) **c, d***P.scaphaner* Szymczakowski, Gunung Mulu (NHMUK) **e, f***P.muluensis* sp. n. Gunung Mulu, paratype (NHMUK) **g, h***P.burckhardti* Schilthuizen & Perreau, Gunung Kinabalu, holotype (MHNG) **i, j***P.sabahensis* Schilthuizen & Perreau, Gunung Kinabalu, holotype (MHNG).

Figures 5e , 7a, b , 9e , 10c

##### Material.

**Holotype**: Malaysia, Sabah, Mt. Kinabalu National Park, Bukit Ular Trail (low), 1800 m elev., multistratum evergreen forest, 2 fish traps, 07–11.xi.1987 (leg. Krikken & Rombaut, RMNH.INS.1086160), male. **Paratypes**: Sabah. Mt. Kinabalu National Park, Bukit Ular Trail (low), 1800 m elev., multistratum evergreen forest, 2 fish traps, 07–11.xi.1987 (leg. Krikken & Rombaut, RMNH.INS.1086161–1086164), 4 females.

##### Description.

Large (3.3–4.0 mm), winged. Elytra, legs and basis and tip of the antennae dark reddish brown, pronotum, head and antennomeres 5–10 nearly black. Pronotum 1.6 times as wide as long, caudal angles clearly extended, as wide as the elytra at the shoulders. Elytra slender, slightly convex, 1.5 times as long as wide (length measured from the caudal tip of the scutellum to the apex of the elytra). Elytral apex in the female gradually rounded and joining the suture at a right angle; in the male more acute, meeting the suture at a sharp angle, and with a distinct bunch of thick, black, outwardly-curved, spine-like setae. Wings present. Body otherwise covered with dense light grey setation. Male tarsi strongly dilated, tarsomeres 1–4 jointly twice as long as wide. Female tarsi not dilated. Aedeagus very strongly curved ventrad, very convex, with a distinct dorsal keel; apex trilobate; stylet very long and thin, hair-like. Spermatheca semicircular, spermiduct extremely long (in extended condition probably at least 5 mm), consisting of ca. 30 360° loops.

##### Differential diagnosis.

Among Sabah *Ptomaphaginus*, and more generally, very distinctive by its large size, dark colouration (but see below under Remarks), the spine-like setae on the male elytral apex, and the uniquely shaped aedeagus (the basic design of which, with two lateral flaps at the apex, resembles that of the *P.bryanti* group, as well as non-Bornean species like *P.sinuatus* Schilthuizen, 1984). The conspicuous bunch of spines at the elytral apex in the male is shared with three other non-Bornean species, viz. *P.riedeli* Perreau, 1995, *P.pilipennis* Perreau, 1991 and *P.pilipennoides* Perreau, 1991, which, however, differ strongly from *P.isabellarossellini* in aedeagal shape. In other Bornean species, *P.bryantioides* and *P.similipes*, stronger setae at the male elytral apex can also be discerned, but never as conspicuous as in *P.isabellarossellini*.

##### Habitat and distribution.

Only known from the lower montane forest around Kinabalu Park Headquarters.

##### Remarks.

It should be noted that specimens of the normally rusty-coloured *P.scaphaner* Szymczakowski, 1972 from the same collection sample as *P.isabellarossellini* are also nearly black. This may mean that the dark colouration of *P.isabellarossellini* is a preservation artefact.

##### Etymology.

Named in honour of the actress and biologist Isabella Rossellini, whose short movies and stage performances on animal reproduction have popularized theories on the evolution of genitalia. In *P.isabellarossellini*, the extremely long penis stylet in the male and similarly long spermiduct in the female suggest a long history of sexually antagonistic coevolution, one of the types of selection that appears in Rossellini’s ‘Green Porno’ series on SundanceTV (Schilthuizen 2014).

**Figure 8.**
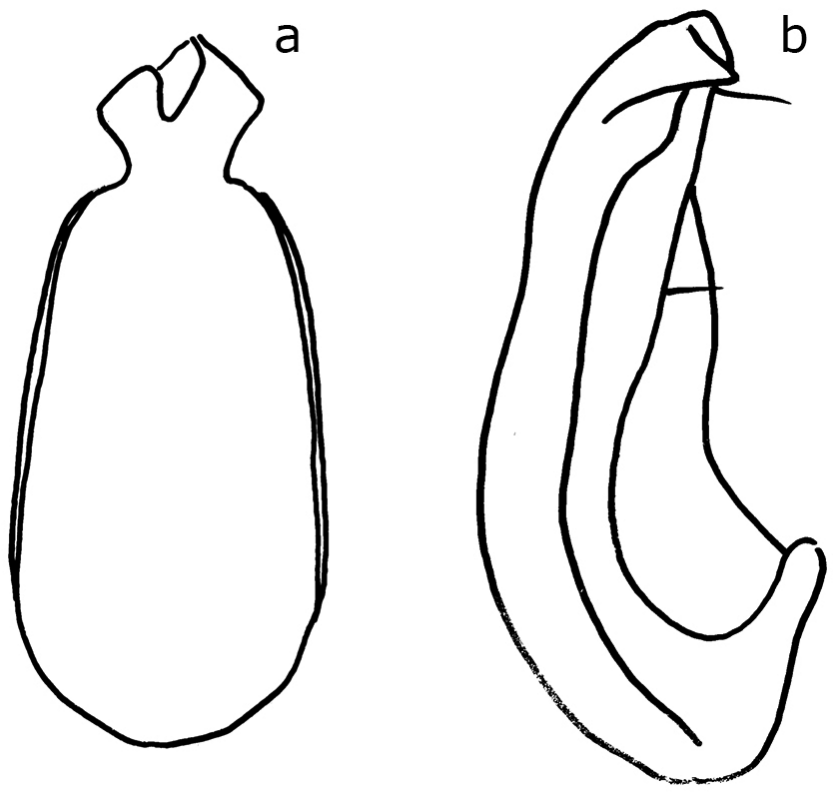
*Ptomaphaginuscaroli*, aedeagus, Crocker Range, holotype (RMNH). **a** dorsal view **b** lateral view (after Schilthuizen & Perreau, 2008). Scale line absent as the drawing is based on a rough sketch made before the aedeagus of the holotype was lost; see text.

#### Ptomaphaginus kinabaluensis

Taxon classificationAnimaliaColeopteraLeiodidae

Schilthuizen & Perreau, 2008

**Figure 9.**
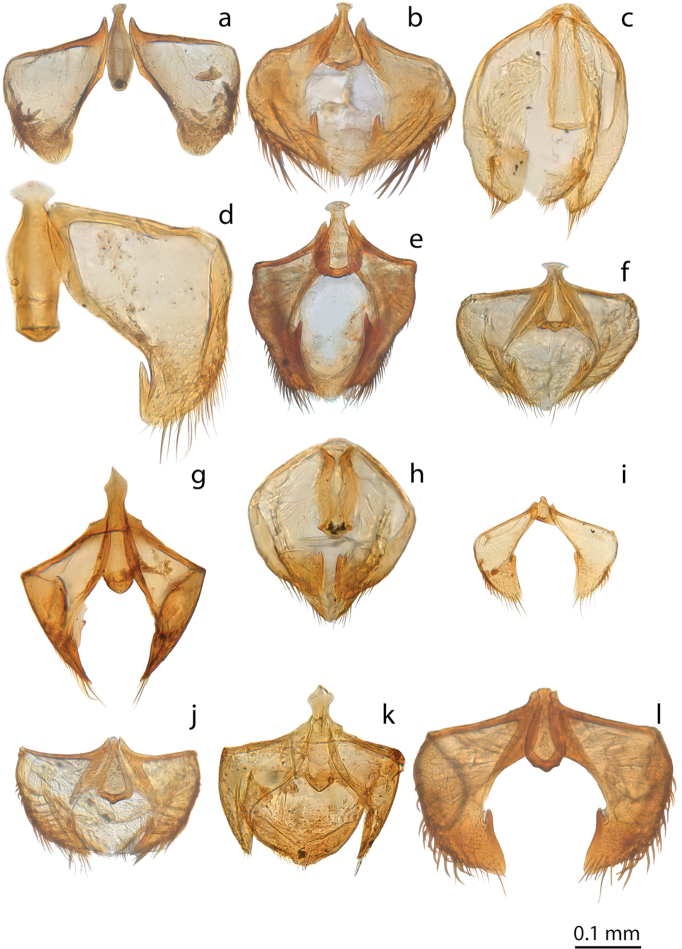
*Ptomaphaginus*, male genital segment (urite IX), dorsal view. **a***P.anas* Schilthuizen & Perreau, Ulu Gombak (RMNH) **b***P.bryantioides* Schilthuizen & Perreau **c***P.burckhardti* Schilthuizen & Perreau, Gunung Kinabalu, holotype (MHNG) **d***P.grandis* sp. n., Gunung Mulu, paratype (NHMUK), left pleurite missing **e***P.isabellarossellini* sp. n., Gunung Kinabalu, holotype (RMNH.INS.1086160) **f***P.kinabaluensis* Schilthuizen & Perreau, Gunung Kinabalu, paratype (RMNH.INS.63302) **g***P.muluensis* sp. n., Gunung Mulu, paratype (NHMUK) **h***P.sabahensis* Schilthuizen & Perreau, Gunung Kinabalu, holotype (MHNG) **i***P.scaphaner* Szymczakowski, Gunung Mulu (NHMUK) **j***P.latimanus* Schilthuizen & Perreau, Gunung Trusmadi (RMNH) **k***P.louis* sp. n., Gunung Mulu, paratype (NHMUK) **l***P.similipes* Schilthuizen & Perreau, Gunung Mulu (NHMUK). Scale bar represents 0.05 mm for Figure 9e.

**Figure 10.**
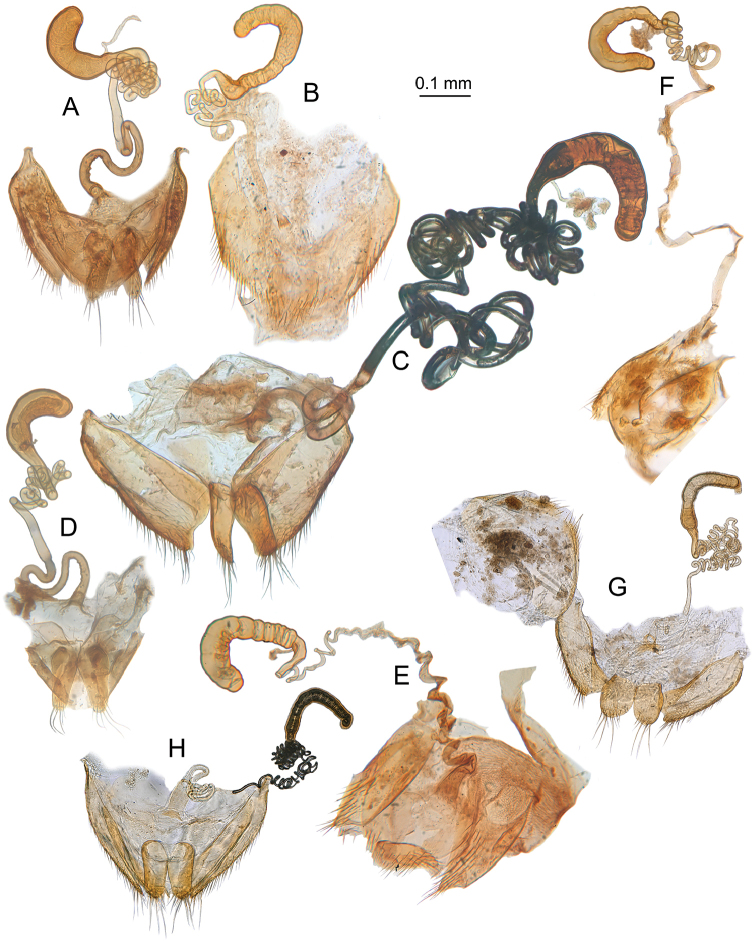
*Ptomaphaginus*, female spermiduct and spermatheca. **a***P.bryantioides* Schilthuizen & Perreau, Gunung Mulu (NHMUK) **b***P.grandis* sp. n., Gunung Mulu, paratype (NHMUK) **c***P.isabellarossellini* sp. n., Gunung Kinabalu, paratype (RMNH.INS.1086161) **d***P.louis* sp. n., Gunung Mulu, paratype (NHMUK) **e***P.muluensis* sp. n., Gunung Mulu, paratype (NHMUK) **f***P.scaphaner* Szymczakowski, Gunung Mulu (NHMUK) **g***P.kinabaluensis* Schilthuizen & Perreau, Gunung Kinabalu (TXEX) **h***P.latimanus* Schilthuizen & Perreau, Gunung Trus Madi, paratype (RMNH).

Figures 4e–f , 6a–b , 9f , 10g

Ptomaphaginus kinabaluensis Schilthuizen & Perreau, 2008: 195, figs 24–25, 29–30; type from Gunung Kinabalu, Sabah, Borneo (in RMNH).

##### Material.

(In addition to that given in Schilthuizen and Perreau (2008)): *Sabah.* Kinabalu Park, HQ, 1540 m elev., pitfall with Limburg cheese, 10–14.ix.2012 (leg. M. Schilthuizen, Crocker Range / Kinabalu Expedition, RMNH.INS.555601–555606), 6 males; Kinabalu Park, HQ, 2003 (leg. Ng Kok Kit, RMNH.INS.63302, 63299, 549265–549267), 5 individuals; Kinabalu Park, Silau Silau (low), 1530 m elev., human excrement traps, 7–11.xi.1987 (leg. Krikken & Rombaut, RMNH.INS.1086165–1086194), 15 males, 15 females; Kinabalu Park, Silau Silau trail (high), 1600 m elev., human excrement trap, 12–17.i.1986 (leg. J. Krikken, RMNH.INS.1086195), 1 female; Kinabalu Park, Bukit Ular trail (low), 1800 m elev., human excrement traps, 7–11.xi.1987 (leg. Krikken & Rombaut, RMNH.INS.1086196–1086197), 2 females; Kinabalu Park, Mempening trail, 1700 m elev., fish traps, 15–22.i.1986 (leg. J. Krikken, RMNH.INS.1086198), 1 male; Kinabalu Park, Mempening trail (high), 1700 m elev., human excrement traps, 7–11.xi.1987 (leg. Krikken & Rombaut, RMNH.INS.1086199), 1 female; Kinabalu Park, Silau Silau (canteen slope), 1540 m elev., fish traps, 16–23.i.1986 (leg. J. Krikken, RMNH.INS.1086200), 1 male; Kinabalu Park, Tempat Pelandok, 1650 m elev., human excrement traps, 9–11.xi.1987 (leg. Krikken & Rombaut, RMNH.INS.1086201–1086204), 1 male, 3 females; Crocker Range Park, Gunung Alab, 1930 m elev., pitfall with Limburg cheese, 17–22.ix.2012 (leg. M. Schilthuizen, Crocker Range / Kinabalu Expedition, RMNH.INS.555629–555631), 2 males, 1 female.

##### Description.

Length 2.3–3.0 mm. Habitus slender, ovoid. Pronotum 1.68–1.86 times as wide as long, as wide as the elytra. Elytra 1.2–1.3 times as long as their combined width (length measured from the caudal tip of the scutellum to the apex of the elytra). Winged. Aedeagus short and wide, with two elongated apical, laterally directed ‘wings’ and a short terminal processus. Spermatheca thin, broadly bent over a 90° angle, slightly bulbous at the basis and with several indistinct annulations at the terminus. Spermiduct long, narrow, with numerous coils. Antennae short, as long as the width of the head. Long setae on the ventral side of the male profemur and protibia absent. Female elytral apices drawn out, male elytral apices rounded, not truncated. Male with a central extension on the 4^th^ visible abdominal sternite.

##### Differential diagnosis.

Similar in aedeagal shape to *P.bryantioides*, but very different in habitus, which is more slender in *P.kinabaluensis*. Furthermore, *P.kinabaluensis* has only slightly dilated male protarsi, no setae on the male profemur and protibia, and extended elytral apices in the female.

##### DNA barcodes.

For the following specimens, DNA barcodes are available in BOLD: RMNH.INS.555601–555606 (Kinabalu Park HQ), RMNH.INS.555629, 555631 (Gunung Alab). RMNH.INS.549265–549267 are available in BOLD, but we have not yet succeeded in obtaining DNA sequences for them.

##### Habitat and distribution.

In montane forest at 1400–1930 m. Sabah: Kinabalu Park (around Park HQ), Crocker Range Park (Gunung Alab and km 51 KK-Tambunan road). The Crocker Range and Kinabalu populations are genetically very similar (BOLD BIN ACK0160).

#### Ptomaphaginus latimanus

Taxon classificationAnimaliaColeopteraLeiodidae

Schilthuizen & Perreau, 2008

Figures 5i , 6g, h , 9j , 10h

Ptomaphaginus latimanus Schilthuizen & Perreau, 2008: 196, figs 22–23; type from Gunung Trus Madi, Sabah, Borneo (in RMNH, RMNH.INS.1086293).

##### Description.

(Adapted from Schilthuizen and Perreau (2008)). Length 2.3–2.9 mm. Habitus slender, ovoid. Pronotum 1.60–1.75 times as wide as long, as wide as the elytra. Elytra 1.15–1.25 times as long as their combined width (length measured from the caudal tip of the scutellum to the apex of the elytra). Winged. Aedeagus short and wide, with two elongated apical, laterally-directed ‘wings’ and a short terminal processus. Spermatheca narrow, annulated, and bent over a rounded 90° angle, quite similar to that of *P.kinabaluensis*. Spermiduct long and narrow, with numerous coils. Antennae short, as long as the width of the head. Long setae on the ventral side of the male profemur and protibia absent. Male with broad and indistinct central notches on the 5^th^ and 6^th^ visible abdominal sternite. Male protarsi strongly dilated.

##### Differential diagnosis.

*Ptomaphaginuslatimanus* is closely related to *P.kinabaluensis*, but differs in the habitus, which is much more stocky in *P.latimanus*. Also, *P.kinabaluensis* has extended elytral apices in the female, less strongly dilated male protarsi, and a central extension on the male 4^th^ abdominal sternite.

##### Habitat and distribution.

Only known from montane forest at Gunung Trusmadi in Sabah, at 1400 m elev. One *bryanti*-group female (RMNH.INS.555611) from Sayap substation on Gunung Kinabalu is genetically unique (BOLD BIN: ACK0183) and might also belong to this species.

#### Ptomaphaginus louis

Taxon classificationAnimaliaColeopteraLeiodidae

Schilthuizen & Perreau sp. n.

http://zoobank.org/67975FC1-077B-4348-86EA-159B147E148C

Figure 5a , 6m, n , 9k , 10d

##### Material.

**Holotype**: Malaysia, Sarawak, Mulu National Park, TPS 7–13, 4.v.1978 (leg. P.M. Hammond & J.E. Marshall, NHMUK, B.M. 1978–49), male. **Paratypes**: *Sarawak.* Mulu National Park, TPS 7–13, 4.v.1978 (leg. P.M. Hammond & J.E. Marshall, NHMUK, B.M. 1978–49), 12 males, 14 females; Mulu National Park, Kerangas?, 7.iv.1978 (leg. P.M. Hammond & J.E. Marshall, NHMUK, B.M. 1978–49), 4 males, 2 females; Mulu National Park, mixed dipterocarp forest litter, TPS 1–2, iv–viii.1978 (leg. P.M. Hammond & J.E. Marshall, NHMUK, B.M. 1978–49), 3 males, 4 females; Mulu National Park, slope, baited traps, 7.iv.1978 (leg. P.M. Hammond & J.E. Marshall, NHMUK, B.M. 1978–49), 1 male, 1 female; Mulu National Park, mixed dipterocarp forest litter, TPS 3–4, v–viii.1978 (leg. P.M. Hammond & J.E. Marshall, NHMUK, B.M. 1978–49), 2 females; Mulu National Park, mixed dipterocarp forest litter, TPS 7–10, v–viii.1978 (leg. P.M. Hammond & J.E. Marshall, NHMUK, B.M. 1978–49), 1 female; Mulu National Park, alluvial forest 100 m, iii–v.1978 (leg. I. Hanski, NHMUK, B.M. 1978–524), 5 females; Mulu National Park, Limestone 6.4, Tp 113, 400 m, 6.iv.1978 (leg. P.M. Hammond & J.E. Marshall, NHMUK, B.M. 1978–49), 1 female; Mulu National Park, mixed dipterocarp forest litter Tp 7, 21.iv.1978 (leg. P.M. Hammond & J.E. Marshall, NHMUK, B.M. 1978–49), 1 male. **Other examined material (not included in the type series)**: *Sarawak.* Mulu National Park, slope, baited traps, 7.iv.1978 (leg. P.M. Hammond & J.E. Marshall, B.M. 1978–49, NHMUK), 2 females.

##### Description.

Length 2.0–2.8 mm. Habitus: Light to dark reddish brown; flattened and relatively short and broad, head broad, pronotum 1.6–1.7 times as wide as long, narrower than the elytra, caudal angles almost not extended. Elytra short, gently convex, jointly ca. 1.2 times as long as wide (length measured from the caudal tip of the scutellum to the apex of the elytra). Body entirely covered in dense golden-yellow setation. Wings present. Antennae slender, 4^th^, 9^th^, and 10^th^ antennomeres almost as long as wide. Male protarsi slightly dilated, the first four tarsomeres jointly ca. 3.5 times as long as wide. Aedeagus gently bent ventrad, flattened, apically broad and convex but subapically tapering in a rounded fashion into a broad but sharp upturned tip. Stylet long and thin, hair-like; stored in a wide loop in the basal part of the aedeagus. *Spiculum gastrale* elongate-ovoid, with the caudal part button-shaped, truncated. Spermatheca semicircular, thick, otherwise featureless, “sausage-shaped”. Spermiduct long, thin, consisting of ca. 4–6 360° coils.

##### Differential diagnosis.

Aedeagus in dorsal view very similar to that of *P.tarsalis* Symczakowski, 1964 from Sumatra, but in lateral view apically clearly more convex and with a shorter stylet. Moreover, the habitus and appendages of *P.tarsalis* are very stout and thick, whereas those in *P.louis* are much more slender. Also very similar to *P.muluensis*, but externally distinguished by the smaller size and more stocky habitus, with shorter elytra, narrower pronotum and the absence of drawn-out caudal pronotal angles. Aedeagus in dorsal view tapering abruptly towards the apex, not as gradually as in *P.muluensis*; in lateral view, the apex is more convex. Spermatheca distinguished from *P.muluensis* by the semicircular shape without any distinctive rings.

##### Habitat and distribution.

Only known from Mulu National Park, Sarawak.

##### Remarks.

Two females from the Mulu locality “Slope” have several rings at the basis of the spermatheca. As they are externally identical to other females of this species, they have been provisionally included in this species, but excluded from the type series. Several specimens infected on the elytra and pygidium with black Laboulbeniales.

##### Etymology.

We name this species after our friend and colleague Dr. Louis Deharveng (MNHN), in recognition for his logistic and emotional support during the preparation of this paper. The specific epithet is given as a noun in apposition.

#### Ptomaphaginus muluensis

Taxon classificationAnimaliaColeopteraLeiodidae

Schilthuizen & Perreau sp. n.

http://zoobank.org/0A383A2B-9730-409A-98F0-7EAAF851B15F

Figures 5b , 7e, f , 9g , 10e

##### Material.

**Holotype**: Malaysia, Sarawak, Mulu National Park, Mixed dipterocarp forest, TPS 7–10, v–viii.1978 (leg. P.M. Hammond & J.E. Marshall, NHMUK, B.M. 1978–49), male. **Paratypes**: *Sarawak*. Mulu National Park, mixed dipterocarp forest, TPS 7–10, v–viii.1978 (leg. P.M. Hammond & J.E. Marshall, NHMUK, B.M. 1978–49), 4 males, 5 females; Mulu National Park, Slope, TPS 13–16, v–viii.1978 (leg. P.M. Hammond & J.E. Marshall, NHMUK, B.M. 1978–49), 9 males, 6 females; Mulu National Park, Slope, TPS 17–21, v–viii.1978 (leg. P.M. Hammond & J.E. Marshall, NHMUK, B.M. 1978–49), 4 males, 5 females; Mulu National Park, Slope, TPS 5–6, v–viii.1978 (leg. P.M. Hammond & J.E. Marshall, NHMUK, B.M. 1978–49), 1 male.

##### Description.

Length 2.5–3.1 mm. Habitus: reddish brown; relatively parallel-sided and somewhat flattened, head broad, pronotum 1.6 times as wide as long, as wide as the elytra, caudal angles clearly extended. Elytra of moderate length, gently convex, in the caudal one-third gently rounded towards the apex, jointly ca. 1.4 times as long as wide (length measured from the caudal tip of the scutellum to the apex of the elytra). Body entirely covered in dense golden-yellow setation. Wings present. Antennae slender, 4^th^, 9^th^, and 10^th^ antennomeres slightly wider than long. Male protarsi slightly dilated, the first four tarsomeres jointly ca. three times as long as wide. Aedeagus gently bent ventrad, in lateral view apically flattened and ending in a bulbous upturned tip, in dorsal view gradually narrowing towards the apex. Stylet long and thin, hair-like. *Spiculum gastrale* long-ovoid, with the caudal part truncated. Spermatheca J-shaped, with 7–10 narrow rings on the long shaft of the “J”, and 5–7 broader rings on the curved part. Spermiduct long, thin, consisting of at least ten 360° coils.

##### Differential diagnosis.

Very similar to *P.louis*, but externally distinguished by the larger size and more slender habitus, with longer elytra, broader pronotum and distinctly drawn-out caudal pronotal angles. Aedeagus in dorsal view tapering gradually towards the apex, not as abruptly as in *P.louis*; in lateral view, the apex is not as convex. Spermatheca distinguished from *P.louis* by the J-shape with distinctive rings.

##### Habitat and distribution.

Only known from Mulu National Park, Sarawak. Several specimens infected with black Laboulbeniales on the elytra and the pygidium.

##### Etymology.

Named after Mulu National Park, to date the only locality from which this species is known.

#### Ptomaphaginus sabahensis

Taxon classificationAnimaliaColeopteraLeiodidae

Schilthuizen & Perreau, 2008

Figures 5g , 7i, j , 9h

Ptomaphaginus sabahensis Schilthuizen & Perreau, 2008: 202, figs 11, 12; type from Gunung Kinabalu, Sabah, Borneo (in MHNG).

##### Description.

(adapted from Schilthuizen and Perreau (2008)). Length 2.4 mm. Habitus elongated, parallel-sided. Pronotum short, 2.3 times as wide as long, nearly as wide as the elytra. Elytra 1.35 times as long as their combined width. Aedeagus quadrangular with a sinuous apical expansion. Internal stylus short and moderately thick. Female unknown.

##### Differential diagnosis.

The elongated habitus combined with the rectangular and stocky aedeagus are unique features among the Bornean *Ptomaphaginus*.

##### Habitat and distribution.

Only known from the male holotype, collected on Gunung Kinabalu at 1580 m elevation.

#### Ptomaphaginus scaphaner

Taxon classificationAnimaliaColeopteraLeiodidae

Szymczakowski, 1972

Figures 5c , 7c, d , 9i , 10f

Ptomaphaginus scaphaner Szymczakowski, 1972: 279–300, figs 28–33); type from Cue phuong Ninh binh, Vietnam (in HNHM).

##### Material.

(in addition to that given in Schilthuizen and Perreau (2008)): *Sabah.* Kinabalu Park, Silau-Silau (low), 1530 m elev., human excrement traps, 7–11.xi.1987 (leg. Krikken & Rombaut, RMNH.INS.1086209–1086214), 5 males, 1 female; Kinabalu Park, Bukit Ular trail (low), 1800 m elev., human excrement traps, 7–11.xi.1987 (leg. Krikken & Rombaut, RMNH.INS.1086224–1086231), 3 males, 5 females; Kinabalu Park, Mempening trail (high), 1700 m elev., human excrement traps, 7–11.xi.1987 (leg. Krikken & Rombaut, RMNH.INS.1086233), 1 male; Crocker Range, Kota Kinabalu-Tambunan road (km 56), 1350 m elev., fish traps, 21–24.xi.1987 (leg. Krikken & Rombaut, RMNH.INS.1086215–1086223), 2 males, 7 females; Kinabalu Park, Headquarters Area, Liwagu Trail, 6°00'29.2"N 116°32'43.8"E, pitfall with chicken, 2.iv.2016 (leg. M. Schilthuizen, I. Njunjić & F. Feijen) 2 males (BORN), 1 male and 1 female (RMNH.INS.1086232). *Sarawak*. Mulu National Park, Limestone 6.4, Tp 113, 400 m elev., 6.iv.1978 (leg. P.M. Hammond & J.E. Marshall, B.M. 1978–49, NHMUK), 1 male; Mulu National Park, Limestone 6.4, Tp 110, 650 m elev., 6.iv.1978 (leg. P.M. Hammond & J.E. Marshall, B.M. 1978–49, NHMUK), 1 male, 2 females; Mulu National Park, mixed dipterocarp forest litter, TPS 5–6, v–viii.1978 (leg. P.M. Hammond & J.E. Marshall, B.M. 1978–49, NHMUK), 4 males, 1 female; Mulu National Park, mixed dipterocarp forest litter, Tp 7, 21.iv.1978 (leg. P.M. Hammond & J.E. Marshall, B.M. 1978–49, NHMUK), 1 male; Mulu National Park, slope, baited traps, 7.iv.1978 (leg. P.M. Hammond & J.E. Marshall, B.M. 1978–49, NHMUK), 3 females; Mulu National Park, slope, TPS 4–6, 29.iv.1978 (leg. P.M. Hammond & J.E. Marshall, B.M. 1978–49, NHMUK), 1 female; Mulu National Park, TPS 7–13, 4.v.1978 (leg. P.M. Hammond & J.E. Marshall, B.M. 1978–49, NHMUK), 1 female.

##### Description.

Length 1.8–2.5 mm. Habitus broad and short, colouration light reddish-brown. Pronotum 1.75 times as wide as long, slightly narrower than the elytra. Elytra 1.2 times as long as jointly wide (length measured from the caudal tip of the scutellum to the caudal tip of the elytra). Wings present. Antennae very broad and short, with antennomeres 9 and 10 twice as broad as long. Aedeagus strongly curved, extremely convex and swollen, ending in a flattened “beak”; stylet long and thin, hair-like, running along the inside of the roof of the convex part of the aedeagus. *Spiculum gastrale* short, triangular, as long as wide. Spermatheca U-shaped, with 6–7 broad rings along the proximal part, and ca. 5 much narrower rings in the terminal one-quarter. Spermiduct very long and very thin.

##### Differential diagnosis.

Unique among the Bornean *Ptomaphaginus* by the condensed antennae and the inflated aedeagus with flattened “beak” and long stylet.

##### Habitat and distribution.

This appears to be a very widespread species, now known from Vietnam, Peninsular Malaysia, Borneo, and Java. It is possible, however, that the species consists of a complex of closely related species, given the geographic variation in secondary sexual characters (Schilthuizen and Perreau 2008).

#### Ptomaphaginus similipes

Taxon classificationAnimaliaColeopteraLeiodidae

Schilthuizen & Perreau, 2008

Figures 5d , 6e, f , 9l

Ptomaphaginus similipes Schilthuizen & Perreau, 2008: 193–194, figs 18–19, 27–28; type from Crocker Range Park, Sabah, Borneo (in RMNH).

##### Material.

(In addition to that given in Schilthuizen and Perreau (2008)): *Sabah.* Kinabalu Park, Sayap substation, 950 m elev., 11–16.ix.2012 (leg. M. Schilthuizen, Crocker Range / Kinabalu Expedition, RMNH.INS.555608–555610, 555612–555614, 555616–555617), 8 individuals; Kinabalu Park, Headquarters Area, Liwagu Trail, 6°00’29.2"N 116°32’43.8"E, pitfall with chicken, 2.iv.2016 (leg. M. Schilthuizen, I. Njunjić & F. Feijen, RMNH.INS.1086234 and BORN), 3 males; Crocker Range, Sugud Forest Reserve, 5°50.361'N, 116°07.084'E, 360 m elev., 1–4.ix.2012 (leg. M. Schilthuizen, Crocker Range / Kinabalu Expedition, RMNH.INS.555592, 555593, 555595), 3 individuals; Crocker Range, Sugud Forest Reserve, 5°50.361'N, 116°07.084'E, 360 m elev., 25.xii.2009 – 01.i.2010 (leg. M. Schilthuizen, RMNH.INS.555678–555685, RMNH.INS.63287–63288, RMNH.INS.549296–549299 and BORN), 14 individuals; Crocker Range, Sugud Forest Reserve, 5°50.361'N, 116°07.084'E, 360 m elev., 10–15.iv.2011 (leg. M. Schilthuizen, RMNH.INS.549263), 1 individual; Crocker Range, Kota Kinabalu-Tambunan road (km 56), 1350 m elev., fish and human excrement traps, 21–24.xi.1987 (leg. Krikken & Rombaut, RMNH.INS.1086239–1086252), 10 males, 8 females; Crocker Range, Keningau-Kimanis road, km 25, 1300 m elev., human excrement traps, 18–23.xi.1987 (leg. Krikken & Rombaut, RMNH.INS.1086235–1086238), 4 individuals. *Sarawak*. Mulu National Park, mixed dipterocarp forest litter, TPS 1–2, v–viii.1978 (leg. P.M. Hammond & J.E. Marshall, B.M. 1978–49, NHMUK), 3 males, 5 females; Mulu National Park, mixed dipterocarp forest litter, TPS 5–6, v–viii.1978 (leg. P.M. Hammond & J.E. Marshall, B.M. 1978–49, NHMUK), 6 males, 8 females; Mulu National Park, mixed dipterocarp forest litter, TPS 7–10, v–viii.1978 (leg. P.M. Hammond & J.E. Marshall, B.M. 1978–49, NHMUK), 4 males, 1 female; Mulu National Park, slope, TPS 4–6, 29.iv.1978 (leg. P.M. Hammond & J.E. Marshall, B.M. 1978–49, NHMUK), 2 males; Mulu National Park, slope, TPS 7–9, 29.iv.1978 (leg. P.M. Hammond & J.E. Marshall, B.M. 1978–49, NHMUK), 2 males, 2 females; Mulu National Park, slope, TPS 10–12, v–viii.1978 (leg. P.M. Hammond & J.E. Marshall, B.M. 1978–49, NHMUK), 2 females; Mulu National Park, slope, TPS 13–16, 29.iv.1978 (leg. P.M. Hammond & J.E. Marshall, B.M. 1978–49, NHMUK), 4 males, 3 females; Mulu National Park, slope, TPS 17–21, 29.iv.1978 (leg. P.M. Hammond & J.E. Marshall, B.M. 1978–49, NHMUK), 2 males; Mulu National Park, slope, baited traps, 7.iv.1978 (leg. P.M. Hammond & J.E. Marshall, B.M. 1978–49, NHMUK), 1 female; Mulu National Park, camp 2, ca. 500 m elev., v–viii.1978 (leg. P.M. Hammond & J.E. Marshall, B.M. 1978–49, NHMUK), 1 female.

##### Description.

Length 2.4–3.0 mm. Pronotum 1.69–1.80 times as wide as long, as wide as the elytra. Elytra 1.19–1.23 times as long as wide (length measured from the caudal tip of the scutellum to the apex of the elytra). Wings present. Aedeagus short and broad, with two short apical, laterally directed ‘wings’ and a short terminal processus. Male forelegs usually with long setae on the ventral side of the femur and tibia. Male protarsus completely not dilated, of the same width as in the female. The 3^rd^, 4^th^, and 5^th^ visible abdominal ventrite of the male carry a slight central notch and show a depression around these notches. Spermatheca narrow, V-shaped, with ca. 10 indistinct rings. Spermiduct long and thin, consisting of ca. 6 360° loops.

##### Differential diagnosis.

Among other members of the *P.bryanti* complex, distinguished by the not dilated male protarsi, thin, annulated spermatheca, the relatively small size, and the short and squat aedeagus.

##### DNA barcodes.

COI barcodes are available for the following specimens: RMNH.INS.63287 (Sugud), RMNH.INS.549263 (Sugud), RMNH.INS.555592–555593 (Sugud), RMNH.INS.555680–555681 (Sugud), RMNH.INS.555685 (Sugud), RMNH.INS.555609–555610 (Sayap), RMNH.INS.555612 (Sayap), RMNH.INS.555616 (Sayap). For RMNH.INS.63288, only the 3’ section of COI is available. For the specimens RMNH.INS.549296–549299 there are entries in BOLD, but we have not yet been able to extract amplifiable DNA from these.

##### Habitat and distribution.

Appears to be widespread on the west coast of Sabah and Sarawak, at elevations between 350 and 1400 m. Some deep molecular divergences are apparent: the specimens collected at Sayap fall in a different DNA barcode BIN than the ones from Sugud (BOLDBINsACK0140 and ACK0141+ABV4636).

##### Remarks.

The male protarsi were slightly dilated in the one male from the Kinabalu Headquarters area.

#### Genus: Ptomaphaminus

Taxon classificationAnimaliaColeopteraLeiodidae

Perreau, 2000

##### Notes.

During the study of this Bornean material, we have refined our concept of the genus *Ptomaphaminus*, which is why the genus description below is more extensive than for the previous genera.

##### Description.

Species of small size, not exceeding 2 mm. Colour generally brown, partly yellowish or light brown, rarely darker. Dorsal surface covered with short recumbent setae inserted along transverse strigae which also cover the whole dorsum of the body. Head with more or less developed eyes. A significant eye reduction is observed in species living in subterranean environments. Antennae generally slender, the apical club weakly marked. Pronotum transverse, the largest width generally at the base, the sides of the pronotum and the elytra continuously arcuate, of equal width. Elytra generally elongate, the posterior sutural angles rounded in males (Figure 14a), in females either rounded (Figure 14b), or simply angular (Figure 14c), or sharply protruding backwards (Figure 14d–e). Surface of elytra with transverse oblique strigae and a single longitudinal sutural stria. Flight wings generally present and functional, even in species living in subterranean environments. (The few apterous or brachypterous species of the genus do not live in Borneo.) Mesoventral process low, narrow, and rounded. Metasternal sutures incomplete and convergent towards the central axis of the body. Protibiae with a lateral row of spines along the external edge and with smaller spines randomly dispersed on the ventral side. Mesotibiae and metatibiae with a circular row of spines around the apex. Male protarsi with four protarsomeres expanded, female protarsi unexpanded. Male and female mesotarsi and metatarsi unexpanded. Male genital segment (urite IX) with a long *spiculum gastrale*, significantly extending beyond the anterior margin of the epipleurites and sometimes expanded into a paddle shape in the distal half. The size of the aedeagus (relatively to the body length) is highly variable: from 3.0 (*P.latescens*) to 5.7 (*P.marshalli*) times smaller than the body length. The left apical expansion of the median lobe generally (in Borneo species) with a more or less developed, ventrally deflexed and sometimes retroverted apical hook, clearly visible in lateral view, more rarely simple (*P.latescens*, *P.testaceus* sp. n.). Endophallus with a long and weakly sinuate stylus and with a basal symmetric (Figures 12a, 12c) or asymmetric (Figures 12f, h, j, l, m, o, q, s, w, y) loop. When asymmetric, the loop is expanded on the left side in dorsal view (which appears on the right side on pictures which are traditionally illustrated with the caudal side up). Female genital segment either with long gonocoxites, more than three times as long as wide (*P.latescens*: Figure 13b; *P.testaceus* sp. n.: Figure 13d), or gonocoxites reduced to small subsquare sclerites (Figures 13f, h, j, n, p, s, u). Spermatheca generally bilobate, with (*P.ater* Perreau, 2009: Figure 13f) or without an apical sclerified plate. A single lobe, weakly sclerotised and transversally ringed occurs in *P.latescens* (Figure 13b) and *P.testaceus* (Figure 13d). Spermiduct less sclerotised, but generally with a fixed morphology, sometimes wrapped in a helical shape, rarely entirely membranous, without structured morphology (*P.hanskii* sp. n., *P.sarawacensis* sp. n.: Figures 13h–13j).

##### Remarks.

The two species *P.latescens* and *P.testaceus* sp. n. have significantly different morphological characters from other species of *Ptomaphaminus* (not limited to Borneo): a short stylus of the endophallus (limited to half the length of the median lobe) with a symmetric basal loop, female gonocoxites long (more than three times longer than wide), a weakly sclerified spermatheca with a set of transversal rings (similar to structures preventing a collapse under depression, like for the respiratory trachea). Other species have a long stylus developed on most of the length of the aedeagus, female gonocoxites short, sub-square and a more sclerified spermatheca without reinforcing transversal rings. These two species form a distinct species group which possibly represents another genus.

##### Biology.

Little information is available on the biology of *Ptomaphaminus*. Two methods of sampling are successful in obtaining specimens: trapping with pitfall traps baited with meat, cheese, or human excrement (either in epigean or in cave environments) and manual collecting in caves. Species collected in epigean conditions generally have fully developed eyes and flight wings while specimens from caves often have reduced eyes (although presently no anophthalmic *Ptomaphaminus* are known). The eye reduction observed in species recorded from caves is not correlated with the flight wing reduction, in contrast to palaearctic and nearctic subterranean species of Cholevinae (Leptodirini; Ptomaphagus (Adelops)). Flight wings of *P.fagei* Perreau, 2009 and *P.latescens* Szymczakowski, 1964, for example, remain fully functional as observed in Gua Sedepan (Eastern Kalimantan) and caves in the Kinabatangan valley (Sabah) where specimens flew up when lighted by headlamps even in the dark zone deep inside caves. A similar observation was reported by Peck (1981) for *P.chapmani* Peck, 1981.

Species are very similar externally. For each of the species below, we provide only specific diagnoses, without listing any shared generic characters.

#### Ptomaphaminus latescens

Taxon classificationAnimaliaColeopteraLeiodidae

(Szymczakowski, 1964)

Figures 12a, b , 13a, b

Ptomaphaginus latescens Szymczakowski, 1964: 140–144, figs 122–132; type from Cave of Durian, Padang Highlands, Sumatra (in RMNH).

##### Material.

*Sabah*. Kinabalu Park, Poring Hot Springs, 6°02.894'N, 116°14.957'E, 625 m elev., 15–20.ix.2012 (leg. M. Schilthuizen, Crocker Range / Kinabalu Expedition, in RMNH.INS.1086253), 1 female; Kinabalu Park Headquarters, 6°00.394'N, 116°32.654'E, 1540 m elev., 10–14.ix.2012 (leg. M. Schilthuizen, Crocker Range / Kinabalu Expedition, RMNH.INS.555607) 1 male; Tinahas Cave, v.2005 (leg. M. Schilthuizen, in RMNH.INS.1086254), 1 male; Crocker Range, Inobong Station, 5°51.265'N, 116°08.363'E, 500 m elev., 21–23.ix.2012 (leg. M. Schilthuizen, Crocker Range / Kinabalu Expedition, RMNH.INS.555643), 1 male; Crocker Range, Gua Laing, 5°28.701'N, 116°10.517E, 613 m elev. (leg. M. Schilthuizen & I. Njunjić, RMNH.INS.1086255–1086257), 3 females; Lower Kinabatangan, Batu Batangan, Gua Ular, 5°27.566'N, 118°06.126'E, 24–28.ii.2017 (leg. M. Schilthuizen, RMNH.INS.5081737, 5081740, 5081743, BOR/COL/14209–14210), 3 males, 2 females; Lower Kinabatangan, Batu Batangan, Gua Kolam, 5°27.557'E, 118°06.118'E, 24.ii–1.iii.2016 (leg. M. Schilthuizen, RMNH.INS. 5081738, 5081741–5081742, 1086284, BOR/COL/14211–14214, 14217), 6 males, 2 females; Lower Kinabatangan, Batu Batangan, Gua Ikan, 5°27.558'N, 118°05.891'E, 24–28.ii.2016 (leg. M. Schilthuizen, RMNH.INS.5081739) 1 male; Lower Kinabatangan, Batu Batangan, Gua Merayap 5°27.573'N, 118°06.075'E, 24–29.ii.2016 (leg. M. Schilthuizen, BOR/COL/14206), 1 female; Lower Kinabatangan, Batu Batangan, Gua Ikan, 5°27.558'N, 118°05.891'E, 18.iii.2015 (leg. M. Schilthuizen & I. Njunjić, RMNH.INS.1086272–1086283), 3 males, 9 females; Lower Kinabatangan, Batu Batangan, Gua Babi, N 5°27.570'N, 118°06.088'E, 24–28.ii.2016 (leg. M. Schilthuizen, RMNH.INS.1086285–1086287 and BOR/COL/14207–14208), 2 males, 3 females; Lower Kinabatangan, Gua Fico, 5°27.135'N, 118°08.769'E, 1–7.iii.2016 (leg. M. Schilthuizen, BOR/COL/14215–14216), 2 males; Lower Kinabatangan, Gua Fico, 5°27.135'N, 118°08.769'E, 20.iii.2015 (leg. M. Schilthuizen & I. Njunjić, RMNH.INS.1086258–108671), 14 males. *Sarawak*. Bau, Gunung Jambusan, 1.401N 110.191E, 24.ii.1980 (RMNH.INS.549312–549316), 2 males, 3 females; Gunung Mulu, mixed dipterocarp forest litter, v–viii.1978 (leg. P.M. Hammond & J.E. Marshall, NHMUK), 2 males, 2 females; Gunung Mulu, Limestone 6.4 400 m Tp 113, v–viii.1978 (leg. P.M. Hammond & J.E. Marshall, NHMUK), 3 males, 2 females; Gunung Mulu, pitfall-trap fish bait, alluv. for. ca. 100 m, iii–v.1978 (leg. I. Hanski, NHMUK), 1 female; Gunung Mulu, 4.5.78 Tps 7–13, v–viii.1978 (leg. P.M. Hammond & J.E. Marshall, NHMUK), 2 females; Gunung Mulu, Limestone 6.4 650 m Tp 110, v–viii.1978 (leg. P.M. Hammond & J.E. Marshall, NHMUK), 2 females; Gunung Mulu, Slope 7.4.78, baited traps, v–viii.1978 (leg. P.M. Hammond & J.E. Marshall, NHMUK), 1 female. *Sumatra*. Cave of Durian (Padang Highlands), 800 m, from excrements of bats, viii.1924, Ptomaphaginuslatescens Szymczakowski, 1964 ZMAN type COLE.0754.1, ZMA.INS.1229114 (leg. E. Jacobson, RMNH), holotype, male; Cave of Durian (Padang Highlands), 800 m, from excrements of bats, viii.1924 (leg. E. Jacobson, ZMA.INS.1229106, 1229112, 1229124, 1229126, 1229128, 1229135, 1229147–1229149, 1229151, 1229153, 1229154), 12 paratypes.

##### Description.

Length 1.45–1.75 mm. General colour dark brown. Winged. Eyes normally developed. Pronotum 1.56 times as wide as long. Elytra 1.35 times as long as wide. Female sutural angle of elytra expanded backwards into a sharp tooth. Male protarsi 0.5 times as wide as the protibia. Male genital segment with a very elongate and thin *spiculum gastrale* (Figure 13a). Aedeagus three times shorter than the body length, strikingly elongate and parallel-sided (Figure 12b). Apex of the median lobe rounded, with two overlapping apical right and left expansions in dorsal view (Figure 12b), regularly arcuate and without apical hook in lateral view (Figure 12a). Two lateral setae on each side, orthogonal to the plane of the aedeagus. Internal stylus of the endophallus short and strongly sinuate, confined to the apical half of the length of the median lobe. Female with normally developed gonocoxites (Figure 13b). Spermiduct irregularly wound, not clearly helical. Spermatheca weakly sclerotised, the apical capsule with a set of transversal rings and without apical sclerotised plate (Figure 13b).

##### Differential diagnosis.

Larger than most other Bornean *Ptomaphaminus*, with spiniform elytral apices in the female, and a very narrow urite IX in the male; differing from the otherwise similar *P.testaceus* by the darker head and discus of the elytra, as well as slight differences in the aedeagus.

##### DNA barcodes.

COI barcodes are available for the following specimen: RMNH.INS.555607 (Kinabalu Park HQ), BIN: ACJ9972. The specimens RMNH.INS.549312–549316 have entries in BOLD, but we have so far been unable to extract amplifiable DNA from them.

##### Habitat and distribution.

Widely distributed in South East Asia. Indonesia: Sumatra (type locality); Kalimantan (Gunung Marang). Malaysia: Continental Malaysia (Batu Caves in Selangor), Sabah (Kinabalu; Lower Kinabatangan), Sarawak (Gunung Jambusan; Gunung Mulu). It has been found in many caves, but also in forest litter and pitfall traps.

##### Remarks.

Variations can be observed in the curvature of the aedeagus. In the type series from Sumatra, the aedeagus is moderately curved, similarly to the population of Marang Mountains, while the population from Continental Malaysia have a more pronounced curvature. We consider that these differences do not exceed intraspecific variation.

#### Ptomaphaminus testaceus

Taxon classificationAnimaliaColeopteraLeiodidae

Schilthuizen & Perreau sp. n.

http://zoobank.org/B7A6C3B9-48F7-4D19-A217-A47B4B05D6AA

**Figure 11.**
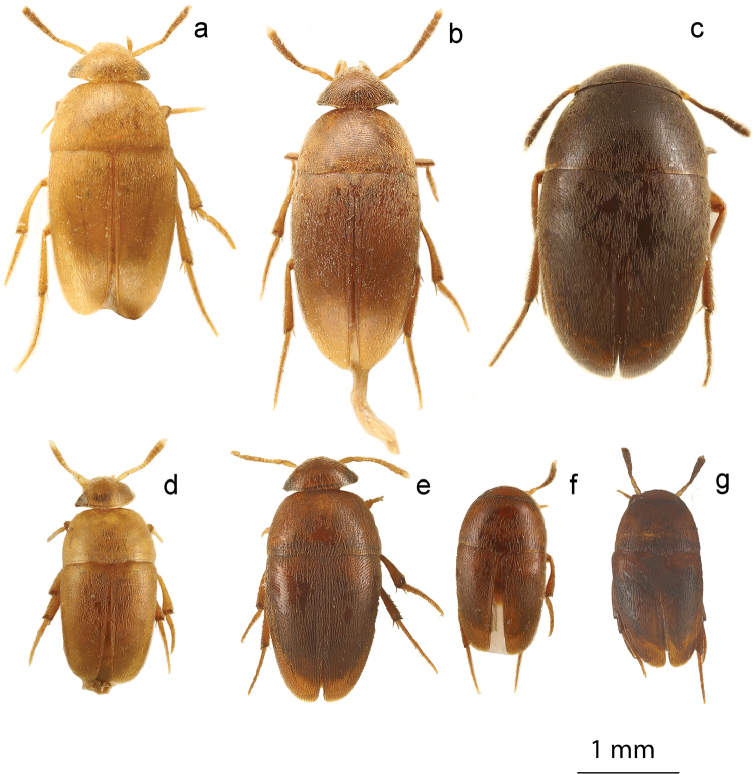
*Ptomaphaminus*, habitus, dorsal. **a–g***P.hanskii*, *sarawacensis*, *layangensis*, *kinabatanganensis*, *testaceus*, *microphallus*, *alabensis*.

**Figure 12.**
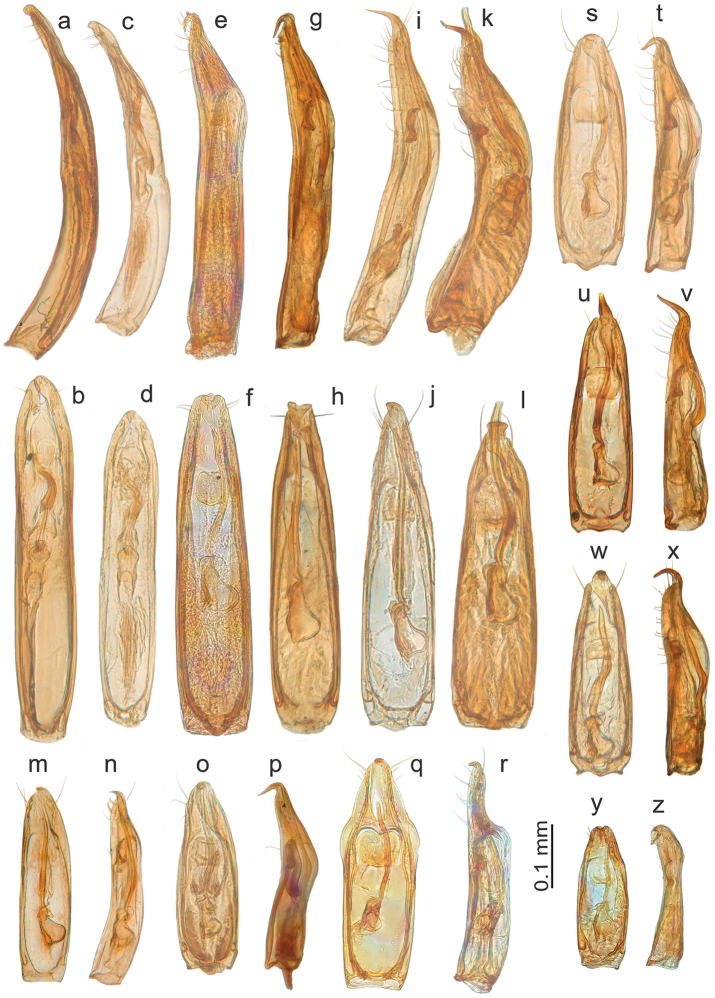
*Ptomaphaminus*, aedeagus. **a, c, e, g, i, k, n, p, r, t, v, x, z** lateral view **b, d, f, h, j, l, m, o, q, s, u, w, y** dorsal view. **a, b***P.latescens***c, d***P.testaceus* sp. n., Sarawak, Gunung Mulu **e, f***P.ater*, paratype (CMPR). **g, h***P.chapmani*, Sarawak, Gunung Mulu **i, j***P.sarawacensis* sp. n., Sarawak, Gunung Mulu **k, l***P.hanskii* sp. n., Sarawak, Gunung Mulu **m, n***P.nanus* sp. n., Sarawak, Gunung Mulu **o, p***P.marshalli* n. sp., Sarawak, Gunung Mulu **q, r***P.fagei* holotype (CMPR) **s, t***P.layangensis* sp. n., Sabah, Gunung Kinabalu, Layang-Layang **u, v***P.kinabatanganensis* sp. n., Sabah, Kinabatangan valley, Gua Ikan **w, x***P.alabensis* sp. n., Sabah, Gunung Alab **y, z***P.microphallus* sp. n., Sabah, Kinabalu, Poring Hot Springs.

**Figure 13.**
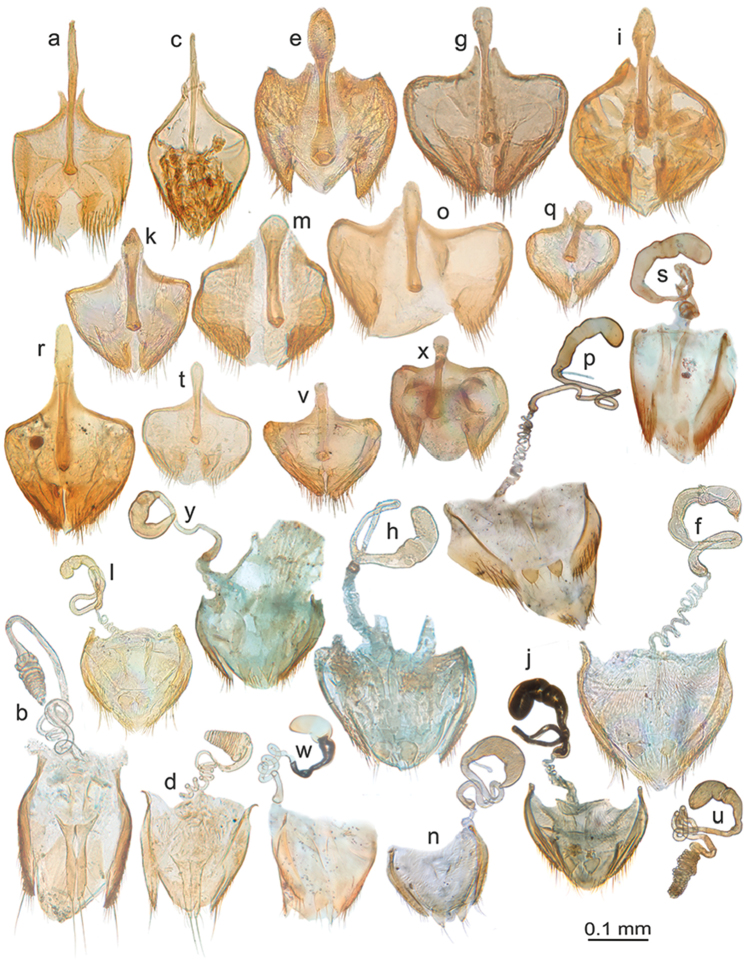
*Ptomaphaminus*, **a, c, e, g, i, k, n, o, r, t, v, x** male genital segments **b, d, f, h, j, l, n, p, q, s, u, w, y** female genital segments. **a, b***P.latescens***c, d***P.testaceus* sp. n. **e, f***P.ater*, paratype (CMPR) **g, h***P.sarawacensis* sp. n., Sarawak, Gunung Mulu **i, j***P.hanskii* sp. n., Sarawak, Gunung Mulu **k, l***P.fagei*, paratype (CMPR) **m, n***P.kinabatanganensis* sp. n. Sabah, Lower Kinabatangan, Gua Fico **o, p***P.layangensis* sp. n. Sabah, Gunung Kinabalu, Layang-Layang **q***P.microphallus* sp. n., Sabah, Kinabalu, Poring Hot Springs **r, s***P.chapmani*, Sarawak, Gunung Mulu **t, u***P.nanus* sp. n., Sarawak, Gunung Mulu. **v, w***P.alabensis* sp. n., Sabah, Gunung Alab. **x, y***P.marshalli* sp. n., Sarawak, Gunung Mulu.

Figures 11e , 12c, d , 13c, d

##### Material.

**Holotype**: Malaysia, Sarawak, 4^th^ Division, Gunung Mulu National Park, mixed dipterocarp forest, v–viii.1978 (leg. P.M. Hammond & J.E. Marshall, NHMUK, B.M. 1978–49), male (aedeagus on separate microscope slide). **Paratypes**: *Sarawak*. Gunung Mulu National Park, pitfall-trap, fish bait, alluv. for., ca. 100 m, iii–v.1978 (leg. I. Hanski, NHMUK, B.M. 1978–524), 2 males, 4 females; Gunung Mulu National Park, mixed dipterocarp forest, v–viii.1978 (leg. P.M. Hammond & J.E. Marshall, NHMUK, B.M. 1978–49), 1 female; Gunung Mulu National Park, 4.5.78, Tps 7–13, v–viii.1978 (leg. P.M. Hammond & J.E. Marshall, NHMUK, B.M. 1978–49), 1 female.

##### Description.

Length: 1.4–1.8 mm. General colour light reddish brown; legs, antenna, and mouthparts yellowish. Winged. Eyes well developed. Pronotum 1.45 times as wide as long. Elytra 1.45 times as long as wide. Female sutural angle of elytra expanded backwards into a sharp tooth. Male protarsi 0.4 times as wide as the apex of protibia. Male genital segment with a very elongate thin *spiculum gastrale* (Figure 13c). Aedeagus approximately 3.25 times shorter than the body length, elongate and parallel-sided, apex rounded, with two overlapping apical right and left expansions in dorsal view (Figure 12d), regularly arcuate from base to apex, without apical hook in lateral view (Figure 12c). Two lateral setae on each side, orthogonal to the plane of the aedeagus. Internal stylus of the endophallus short and strongly sinuate, confined to the apical half of the length of the median lobe (Figure 12c). Female genital segment with normally developed gonocoxites. Spermiduct slightly helical. Spermatheca weakly sclerified, with a set of transverse rings and without apical sclerotised plate (Figure 13d).

##### Differential diagnosis.

Very similar to *P.latescens*, but distinct in the external morphology by its significantly smaller size, and its lighter colour. The spermiduct is slightly helical, which is not the case in *P.latescens*.

##### Habitat and distribution.

Known only from the lowland forest of Gunung Mulu, Sarawak, Malaysia.

##### Etymology.

Named for its light brown colour (*testaceus* = brick-coloured).

#### Ptomaphaminus ater

Taxon classificationAnimaliaColeopteraLeiodidae

Perreau, 2009

**Figure 14.**
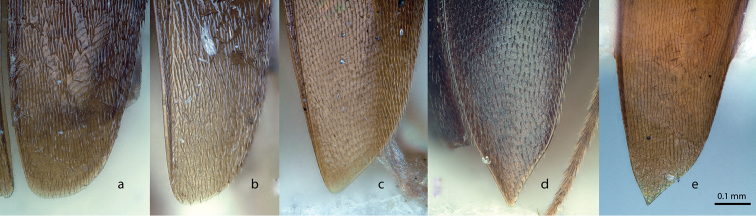
*Ptomaphaminus*, elytral apices. **a***P.marshalli*, male, Sarawak, Gunung Mulu **b***P.marshalli*, female, Sarawak, Gunung Mulu **c***P.sarawacensis*, female Sarawak, Gunung Mulu **d***P.ater* female paratype (CMPR) **e***P.chapmani* female Sarawak, Gunung Mulu.

Figures 12e, f , 13e, f , 14d

Ptomaphaminus ater Perreau, 2009: 5, fig. 5; type from Gunung Kinabalu, Sabah, Borneo (in MHNG).
Ptomaphaginus ater : Merckx et al. 2015: extended data figs 2, 6.

##### Material.

(In addition to that listed in Perreau 2009): *Sabah.* Kinabalu Park, Paka Cave, 3080 m elev., 14–19.ix.2012 (leg. M. Schilthuizen, Crocker Range / Kinabalu Expedition, RMNH), 10 individuals (incl. RMNH.INS.555623–555624, RMNH.INS.1086288–1086290).

##### Description.

Length 1.75–2.00 mm. Large species, dark brown, winged. Eyes reduced, with 25 ommatidia. Pronotum ca. 1.5 times wider than long. Elytra approximately 1.2 times longer than wide. Female apex of the elytra with a sharp sutural angle expanded posteriorly (Figure 14d). Male protarsi approximately 0.6 times as wide as the apex of protibia. Male genital segment with long and apically expanded *spiculum gastrale*, widely dilated into a kind of paddle (Figure 13e). Aedeagus long, 3.5 times shorter than the body length, parallel-sided, straight, shortly narrowed near the apex in dorsal view (Figs 12f), ventrally bent on the last quarter of its length and with an apical hook clearly retroverted ventrally (Figure 12g). Stylet of the endophallus long and straight. Female genital segment with reduced gonocoxites. Spermiduct helical. Spermatheca long, bilobate, rounded at the apex, but with an apical sclerotised plate (Figure 13f).

##### DNA barcodes.

For two individuals, RMNH.INS.555623–555624, COI barcodes are available in BOLD, which form the BIN ACK0013.

##### Differential diagnosis.

Externally recognizable by its large size, dark colouration, and strongly cuneiform habitus. Apex of the female elytra spiniform. Spermatheca with a sclerotised plate at its apex. Aedeagus blunt-ended.

##### Habitat and distribution.

Known from high altitude on Gunung Kinabalu, above 3000 m. Some specimens were taken under a rocky overhang (Paka cave), others in Panar Laban (type locality) and Gunting Lagadan, without detail on collecting conditions.

##### Remarks.

The only species in Borneo with an apical sclerotised plate at the apex of the spermatheca (which occurs in several other species outside Borneo).

#### Ptomaphaminus kinabatanganensis

Taxon classificationAnimaliaColeopteraLeiodidae

Njunjić, Schilthuizen & Perreau sp. n.

http://zoobank.org/ED299A72-768F-4F5E-8EB8-60CDD927ED6F

Figures 11d , 12u, v , 13m, n

##### Material.

**Holotype**: Malaysia, Sabah, Lower Kinabatangan, Batu Batangan, Gua Kolam, 5°27.557'N, 118°06.118'E, 24.ii–1.iii.2016 (leg. I. Njunjić et al., BOR/COL/14218), 1 male. **Paratypes**: *Sabah*. Lower Kinabatangan, Batu Batangan, Gua Babi, 5°27.570'N, 118°06.088'E, 24–28.ii.2016 (leg. field course students, BOR/COL/14219–14220), 2 individuals; Lower Kinabatangan, Gua Fico, 5°27.135'N, 118°08.769'E, 18.iii.2015 (leg. I. Njunjić et al., CMPR), 1 female; Lower Kinabatangan, Batu Batangan, Gua Babi, N 5°27.570'N, 118°06.088'E, 24–28.ii.2016 (leg. I. Njunjić et al., CMPR), 1 female; Lower Kinabatangan, Batu Batangan, Gua Ikan, 5°27.558'N, 118°05.891'E, 18.iii.2015 (leg. I. Njunjić et al., CMPR), 2 males.

##### Description.

Length: 1.45–1.60 mm. General colour brown; antenna, mouthparts, and protarsi yellowish, the other tarsi light brown. Winged. Eyes well developed. Pronotum 1.55 times wider than long. Elytra 1.25 times longer than wide (slightly wider in males than in females). Female sutural apex of elytra angular but not protruding backwards. Male protarsi 0.65 times as wide as the apex of protibia. *Spiculum gastrale* of the male genital segment shortly protruding beyond the apex of epipleurites, significantly dilated (Figure 13m). Aedeagus approximately 4 times smaller than the body length, parallel on the first third of its length, then regularly narrowed, the sides linearly convergent towards the apex in dorsal view (Figure 12u), straight in lateral view (Figure 12v). Apex of the median lobe with a long hook deflexed towards the ventral side, but not retroverted in lateral view (Figure 12v). Six lateroventral preapical setae on each side. Internal stylus of the endophallus long and moderately sinuate. Female genital segment with reduced gonocoxites. Spermiduct not helical. Spermatheca rounded at the apex without sclerotised plate (Figure 13n).

##### Differential diagnosis.

Female with spiniform elytra; male with long apical hook of the (relatively short but straight) aedeagus, which, however, is not retroverted.

##### Habitat and distribution.

Known exclusively from three caves in the lower Kinabatangan valley: Gua Babi, Gua Fico, and Gua Ikan.

##### Etymology.

Named after the lower Kinabatangan valley, in which the specimens were collected.

#### Ptomaphaminus chapmani

Taxon classificationAnimaliaColeopteraLeiodidae

(Peck, 1981)

Figures 12g–h , 13r–s , 14e

Ptomaphaginus chapmani Peck, 1981. Peck (1981: 222, fig. 1–4); type from Mulu, Sarawak, Borneo (in NHMUK).

##### Material.

Sarawak. Mulu, Mayday Cave, rotten prawn bait, 24.vii.1980 (RMNH.INS.634810, 634795), 2 individuals; Mulu, Clearwater Cave, Snake Track passage, mice-rat bait, 5.i.1981 (RMNH.INS.634763, 634819, 634784), 3 individuals; Mulu, Clearwater Cave, 9.v.1978 (leg. P. Chapman, RMNH.INS.634799), 1 paratype; Mulu, Mayday Cave, rotted prawn bait, 24.vii.1980 (CMPR), 7 individuals.

##### Description.

Length 1.40–2.0 mm. Colour light brown. Winged. Eyes with ten ommatidia. Pronotum 1.47 times as wide as long, elytra 1.84 times as long as wide. Female sutural angle of elytra expanded in a sharp apical tooth (Figure 14e). Protarsi 0.8 times as wide as the protibia. *Spiculum gastrale* of the male genital segment moderately dilated in a parallel-sided spatula (Figure 13r). Aedeagus long, approximately 3.3 times smaller than the body length, parallel, weakly arcuate in lateral view and shortly narrowed on the apical quarter of its length, rectangular at the apex, with an apical hook strongly retroverted ventrally (Figure 12g, h). Internal stylus of the endophallus long and straight. Female genital segment with reduced gonocoxites. Spermiduct not helical. Spermatheca rounded at the apex without sclerotised plate (Figure 13s).

##### Differential diagnosis.

Unique among Bornean *Ptomaphaminus* in showing a combination of troglomorphic features: reduced eyes and elongated habitus. Aedeagus very similar to that of *P.ater*, which, however, is darker and has a more strongly cuneiform habitus.

##### Habitat and distribution.

Known only from a single cave (Clearwater cave) in Gunung Mulu, Sarawak, Malaysia.

#### Ptomaphaminus fagei

Taxon classificationAnimaliaColeopteraLeiodidae

Perreau, 2009

Figures 12q–r , 13k–l

Ptomaphaminus fagei Perreau, 2009. Perreau (2009: 2, fig. 4); type from Gunung Marang, Kalimantan, Borneo (in CMPR).

##### Material.

*Kalimantan Timur*. Kebupaten Kutai Timur, karst of Mangkalihat, Mt Marang, Gua Sedepan, 8.vi.2002 (leg. M. Perreau, Expédition du Kalimanthrope, TXEX), 1 male, 1 female paratypes.

##### Description.

Length 1.50–1.90 mm. Colour light brown. Winged. Eyes with 15 ommatidia. Body very elongate, parallel-sided. Pronotum approximately 1.6 times wider than long. Elytra approximately 1.4 times longer than wide. Female sutural angle of elytra expanded backwards in a sharp tooth. Male protarsi 0.75 times as wide as the apex of protibia. *Spiculum gastrale* of the male genital segment expanded, with a triangular apical part (Figure 13k). Aedeagus 4.8 times as long as the body length, parallel-sided at the base, triangularly narrowed in the last third of the length in dorsal view (Figure 12q), thick at the base, abruptly thinned in the two apical third of its length and ending with a short ventrally deflexed hook in lateral view (Figure 12r). Five lateroventral lateral external setae and three more ventral central setae on each side. Internal stylus of the endophallus straight. Parameres with five apical external and three apical internal setae. Female genital segment with reduced gonocoxites, spermatheca bilobate, with helicoidal spermiduct, and without apical sclerotised plate (Figure 13l).

##### Differential diagnosis.

Among the species with similarly reduced eyes and/or spiniform female elytral apices, *P.fagei* is unique in having an aedeagus that shows an abrupt narrowing (in lateral view) in the apical third.

##### Habitat and distribution.

Known from two caves of Gunung Marang, Kalimantan, Indonesia: Gua Sedepan and Gua Gala.

#### Ptomaphaminus nanus

Taxon classificationAnimaliaColeopteraLeiodidae

Schilthuizen & Perreau sp. n.

http://zoobank.org/78CBED84-54FD-4632-89F7-FAA3944D5921

Figures 12m–n , 13t–u

##### Material.

**Holotype**: Malaysia, Sarawak, 4^th^ Division, Gunung Mulu National Park, mixed dipterocarp forest litter, v–viii.1978 (leg. P.M. Hammond & J.E. Marshall, in NHMUK, B.M.1978–49), 1 male. **Paratypes**: *Sarawak*. Gunung Mulu National Park, 4.5.78 Tps 7–13, v–viii.1978 (leg. P.M. Hammond & J.E. Marshall, NHMUK, B.M.1978–49), 7 males, 4 females; Gunung Mulu National Park, mixed dipterocarp forest litter, v–viii.1978 (leg. P.M. Hammond & J.E. Marshall, NHMUK, B.M.1978–49), 2 males; Gunung Mulu National Park, limestone 6.4, 650 m, tp 110, v–viii.1978 (leg. P. E. Hammond & J. E. Marshall, B. M. 1978–49, NHMUK), 1 female.

##### Description.

Length: 1.28–1.50 mm. General colour brown; tarsi, antenna, mouthparts yellowish. Winged. Eyes well developed. Pronotum1.6 times as wide as long. Elytra 1.2 times as long as wide. Elytral internal angle rounded in male and in female, without noticeable sexual dimorphism. Male protarsi 0.6 times as wide as the protibia. *Spiculum gastrale* of the male genital segment dilated into a narrow spatula (Figure 13t). Aedeagus approximately 4.7 times smaller than the body length, slightly arcuate in lateral view and the sides slightly arcuate in dorsal view, the apex with a short ventrally deflexed hook (Figures 12m, 2n). Internal stylus of the endophallus long and nearly straight. On each side one lateroventral preapical seta, one lateroapical, and one seta located at the apical third of the length of the aedeagus. The lateroapical seta is pointing forward, the other orthogonally to the plane of the aedeagus. In addition, there are two very strong preapical setae on each side, which have no equivalent in other species. Female genital segment with reduced gonocoxites. Spermiduct helical with a very large number of tightly compacted coils. Apex of the spermatheca rounded, without apical sclerotised plate (Figure 13u).

##### Differential diagnosis.

Small-sized species with normally developed eyes and non-spiniform female elytra. Spermiduct tightly coiled; aedeagus small; distinguishable from *P.marshalli*, which has an equally small aedeagus, by the very short hook.

##### Habitat and distribution.

Known from lowland forests of Gunung Mulu, Sarawak, Malaysia.

##### Remarks.

A very small species, one of the smallest species of the genus.

##### Etymology.

Named for its very small size (*nanus* = dwarf).

#### Ptomaphaminus marshalli

Taxon classificationAnimaliaColeopteraLeiodidae

Schilthuizen & Perreau sp. n.

http://zoobank.org/B09BB75F-114E-42C4-B3FD-B6490A445EB5

Figures 12o, p , 13x, y , 14a, b

##### Material.

**Holotype**: Malaysia, Sarawak, 4^th^ Division, Gunung Mulu National Park, Slope, 29.4.78, Tps 4–6, v–viii.1978 (leg. P.M. Hammond & J.E. Marshall, in NHMUK, B.M.1978–49), 1 male. **Paratypes**: Sarawak. Gunung Mulu National Park, Slope, 29.4.78, Tps 4–6, v–viii.1978 (leg. P.M. Hammond & J.E. Marshall, NHMUK, B.M.1978–49), 2 males, 1 female; Gunung Mulu National Park, Slope, 29.4.78, Tps 1–3, v–viii.1978 (leg. P.M. Hammond & J.E. Marshall, NHMUK, B.M.1978–49), 6 males, 7 females; Gunung Mulu National Park, Slope, 7.4.78, baited traps, v–viii.1978 (leg. P.M. Hammond & J.E. Marshall, NHMUK, B.M.1978–49), 1 female. *Sabah*. Crocker Range Park, Inobong Station, 5°51.265'N, 116°06.363'E, 500 m elev., 21–23.ix.2012 (leg. M. Schilthuizen, Crocker Range / Kinabalu Expedition, RMNH.INS.555639), 1 male.

##### Description.

Length: 1.50–1.90 mm. General colour brown; the tarsi, the two first antennomeres, the base of the third antennomere, and of the tibiae yellowish. Winged. Eyes well developed. Pronotum 1.65 times as wide as long. Elytra 1.25 times as long as wide. Female sutural apex of elytra rounded (Figure 14b), similar to the male (Figure 14a). *Spiculum gastrale* of the male genital segment apically dilated into a short discoid expansion (Figure 13x). Aedeagus approximately 5.7 times shorter than the body length, the sides regularly arcuate in dorsal view (Figure 12o), moderately arcuate ventrally and obtusely angular dorsally in lateral view (Figure 12p). Apex of the median lobe with a large ventrally deflexed but not retroverted hook. Four lateroventral setae spaced from the apical third of the aedeagus and the apex. Internal stylus of the endophallus moderately sinuate. Female genital segment with reduced gonocoxites. Spermiduct not helical. Apex of the spermatheca conically narrowed, the apex shortly rounded, without apical sclerotised plate (Figure 13y).

##### Differential diagnosis.

Females of this species can be recognized by the apex of the spermatheca, which is conically narrowed, a very distinct morphology compared to all other species, which have a spermatheca with a widely rounded apex.

##### Habitat and distribution.

Known from lowland forests of Gunung Mulu, Sarawak, and of Gunung Kinabalu, Sabah, Malaysia.

##### Etymology.

Dedicated to J. E. Marshall, one of the collectors of the species during the expedition of the Natural History Museum of London in Sarawak.

#### Ptomaphaminus hanskii

Taxon classificationAnimaliaColeopteraLeiodidae

Schilthuizen & Perreau sp. n.

http://zoobank.org/375C731A-B3B0-49DF-A150-51FA4F0D7A41

Figure 11a , 12k, l , 13i, j

##### Material.

**Holotype**: Malaysia, Sarawak, 4^th^ Division, Gunung Mulu National Park, Slope, 4.5.78, 9.1, 9.3, v–viii.1978 (leg. P.M. Hammond & J.E. Marshall, in NHMUK, B.M.1978–49), 1 male. **Paratypes**: *Sarawak*. Gunung Mulu National Park, Slope, 4.5.78, 9.1, 9.3, v–viii.1978 (leg. P.M. Hammond & J.E. Marshall, NHMUK, B.M.1978–49), 5 males, 8 females.

##### Description.

Length: 1.6–2.1 mm. General colour brown; the tarsi and two first antennomeres yellowish. Winged. Eyes well developed. Pronotum 1.65 times as wide as long. Elytra 1.30 times as long as wide. Sutural angle of female elytra angular. Apex of the *spiculum gastrale* of the male genital segment dilated into a diamond-like form (Figure 13i). Aedeagus elongated, approximately 3.5 times shorter than the body length. Median lobe regularly narrowed from base to the apex in dorsal view (Figure 12l), thick and strongly arcuate, with a long ventrally deflexed but not retroverted apical hook in lateral view (Figure 12k). Six lateroventral preapical setae and one lateroapical seta on each side. Internal stylus of the endophallus moderately sinuate. Female genital segment with reduced gonocoxites. Spermiduct membranous, vaguely helical at least near the base of the spermatheca. Apex of the spermatheca rounded, without apical sclerotised plate (Figure 13j).

##### Differential diagnosis.

Species with normally developed eyes and a long aedeagus. Very similar to *P.sarawacensis*, from which it cannot be confidently distinguished in the female sex. Males of *P.hanskii* have a thicker median lobe of the aedeagus than *P.sarawacensis*.

##### Habitat and distribution.

Known from lowland forests of Gunung Mulu, Sarawak, Malaysia.

##### Remarks.

The external morphology and the female genital morphology are extremely similar to *P.sarawacensis*, so that females are nearly impossible to distinguish. However, the male aedeagi of these species are very different.

##### Etymology.

Named in honour of Ilkka Hanski, the Finnish ecologist who played an important role in the Royal Geographical Society expedition to Mulu of 1978, and who passed away in 2016.

#### Ptomaphaminus sarawacensis

Taxon classificationAnimaliaColeopteraLeiodidae

Schilthuizen & Perreau sp. n.

http://zoobank.org/B1280A81-B7FC-48B2-9997-9133EF5B4D85

Figures 11b , 12i, j , 13g, h , 14c

##### Material.

**Holotype**: Malaysia, Sarawak, 4^th^ Division, Gunung Mulu National Park, Slope, 4.5.78, 9.1, 9.3, v–viii.1978 (leg. P.M. Hammond & J.E. Marshall, in NHMUK, B.M.1978–49), 1 male. **Paratypes**: *Sarawak*. Gunung Mulu National Park, Slope, 4.5.78, 9.1, 9.3, v–viii.1978 (leg. P.M. Hammond & J.E. Marshall, NHMUK, B.M.1978–49), 5 males, 12 females.

##### Description.

Length: 1.90–2.35 mm. General colour brown; the tarsi and two first antennomeres yellowish. Winged. Eyes well developed. Pronotum 1.6 times as wide as long. Elytra 1.4 times as long as wide. Sutural angle of female elytra angular (Figure 14c). Apex of the *spiculum gastrale* of the male genital segment dilated into a paddle-like shape (Figure 13g). Aedeagus approximately four times shorter than the body length. Median lobe parallel-sided in the first half, then regularly narrowed from the middle of its length, with a preapical constriction in dorsal view (Figure 12j), obtusely angular ventrally after the middle, with a long apical ventrally deflexed but not retroverted hook in lateral view (Figure 12i). Six lateroventral preapical setae and one lateroapical seta on each sides. Internal stylus of the endophallus nearly straight. Female genital segment with reduced gonocoxites. Spermiduct membranous, vaguely helical at least near the base of the spermatheca. Apex of the spermatheca rounded, without apical sclerotised plate (Figure 13h).

##### Differential diagnosis.

See under *P.hanskii* (above).

##### Habitat and distribution.

Known from lowland forests of Gunung Mulu, Sarawak, Malaysia.

##### Remarks.

Extremely similar to *P.hanskii*, except in aedeagus shape.

##### Etymology.

Named for the Malaysian state of Sarawak, where the type locality lies.

#### Ptomaphaminus layangensis

Taxon classificationAnimaliaColeopteraLeiodidae

Schilthuizen & Perreau sp. n.

http://zoobank.org/A840208F-B55E-486C-887F-A26A62243AEB

Figures 11c , 12s, t , 13o, p

Ptomaphaginus sp. n. bryanti complex Merckx et al., 2015: extended data fig. 6.

##### Material.

**Holotype**: Malaysia, Sabah, Kinabalu Park, Layang-Layang, 2750 m elev., 6°02.748'N, 116°33.632'E, 13–18.ix.2012 (leg. M. Schilthuizen, Crocker Range / Kinabalu Expedition, RMNH.INS.555619), 1 male. **Paratypes**: *Sabah*. Kinabalu Park, Layang-Layang, 6°02.748'N, 116°33.632'E, 2750 m elev., 13–18.ix.2012 (leg. M. Schilthuizen, Crocker Range / Kinabalu Expedition, CMPR, RMNH, incl. RMNH.INS.555618–555622), 11 paratypes.

##### Description.

Length: 1.6–2.5 mm. General colour dark brown; the tarsi and two first antennomeres lighter. Winged. Eyes well developed. Pronotum 1.6 times as wide as long. Elytra 1.45 times as long as wide. Sutural angle of female elytra rounded, without noticeable sexual dimorphism. Male protarsi 0.75 times as wide as the protibia. *Spiculum gastrale* of the male genital segment short, moderately dilated at the apex (Figure 13o). Aedeagus 5.4 times shorter than the body length, the sides regularly arcuate in dorsal view (Figure 12s), ventrally straight and dorsally obtusely angular in lateral view. Apex of the median lobe with a ventrally deflexed, arcuate hook in lateral view (Figure 12t). Six lateroventral preapical setae and one lateroapical seta on each side. Internal stylus of the endophallus significantly sinuate. Female genital segment with reduced gonocoxites. Spermiduct long and helical (Figure 13p). Apex of spermatheca rounded, without apical sclerotised plate.

##### Differential diagnosis.

Normal-sized species with unreduced eyes and non-spiniform female elytra. Aedeagus with a long, retroverted apical hook. Distinguishable from *P.alabensis* by the anteriorly widened *spiculum gastrale* and the broader apex of the median lobe of the aedeagus. Females are distinguishable from *P.nanus* by their larger size.

##### DNA barcodes.

COI barcodes are available in BOLD for the holotype, RMNH.INS.555619, as well as for the paratypes RMNH.INS.555618 and 555620–555622, jointly forming the BIN ACK0070.

##### Habitat and distribution.

Known from high altitude (above 2000 m) on Gunung Kinabalu, Sabah, Malaysia.

##### Etymology.

Named for the type locality on Gunung Kinabalu.

#### Ptomaphaminus microphallus

Taxon classificationAnimaliaColeopteraLeiodidae

Schilthuizen & Perreau sp. n.

http://zoobank.org/F32A3991-EC96-4F68-8C61-464F62432FAF

Figures 11f , 12y, z , 13q

##### Material.

**Holotype**: Malaysia, Sabah, Kinabalu Park, Poring Hot Springs, 6°02.894'N, 116°41.957'E, 625 m elev., in baited pitfall traps, 15–20.ix.2012, (leg. M. Schilthuizen, Crocker Range / Kinabalu Expedition), 1 male in RMNH (RMNH.INS.1086291). **Paratypes**: *Sabah*. Kinabalu Park, Poring Hot Springs, 6°02.894'N, 116°41.957'E, 625 m elev., in baited pitfall traps, 15–20.ix.2012 (leg. M. Schilthuizen, Crocker Range / Kinabalu Expedition), 1 male paratype in RMNH (RMNH.INS.1086292).

##### Description.

Length: 1.2 mm. General colour dark brown; the tarsi and two first antennomeres lighter. Winged. Pronotum 1.55 times as wide as long. Elytra 1.15 times as long as wide. Male protarsi 0.6 times as wide as the protibia. *Spiculum gastrale* of the male genital segment apically dilated into a short discoid expansion (Figure 13q). Aedeagus very small, 5.5 times shorter than the body length. Lateral sides regularly arcuate in dorsal view (Figure 12y), flattened in the middle and with a short ventrally retroverted expansion in lateral view (Figure 12z). On each side, six latero-preapical pointing ventral setae and one apical seta pointing forward. Internal stylus of the aedeagus weakly sinuate.

##### Differential diagnosis.

The female is unknown, but the male aedeagus shares several features with other species, such as *P.marshalli*, *P.nanus*, and *P.alabensis* (i.e., a relatively short aedeagus with retroverted hook). However, *P.microphallus* is unique among Borneo *Ptomaphaminus* by its extremely short (0.22 mm) aedeagus.

##### Habitat and distribution.

Known from the type locality, in Kinabalu Park, Sabah, Malaysia.

##### Remarks.

Female unknown.

##### Etymology.

Named for the relatively small male genitalia.

#### Ptomaphaminus alabensis

Taxon classificationAnimaliaColeopteraLeiodidae

Schilthuizen & Perreau sp. n.

http://zoobank.org/1F9CBD47-54C9-4F92-B7D3-DE35E2DEAF20

Figures 11g , 12w, x , 13v, w

Ptomaphaginus nr. fagei Merckx et al., 2015: extended data figs 2, 6.

##### Material.

**Holotype**: Malaysia, Sabah, Crocker Range, Gunung Alab, 5°47.766'N, 116°20.504'E, 1930 elev., baited pitfall trap, 17–22.iv.2012 (leg. M. Schilthuizen, RMNH.INS.555632)**. Paratype**: *Sabah*. Crocker Range, Gunung Alab, 5°47.766'N, 116°20.504'E, 1930 elev., baited pitfall trap, 17–22.iv.2012 (leg. M. Schilthuizen, Crocker Range / Kinabalu Expedition, RMNH, CMPR), 3 males, 3 females (including RMNH.INS.555633).

##### Description.

Length: 1.5–1.6 mm. General colour dark brown; the tarsi and two first antennomeres yellowish. Winged. Pronotum 1.6 times as wide as long. Elytra 1.33 times as long as wide. Sutural angle of female elytra rounded, without noticeable sexual dimorphism. Male protarsi 0.8 times as wide as the apex of protibia. *Spiculum gastrale* of the male genital segment straight, without apical dilation. Median lobe of the aedeagus 4.7 times shorter than the body length. Apex of the median lobe with a ventrally deflexed, arcuate hook. Aedeagus regularly narrowed from base to apex in dorsal view, ventrally straight (Figure 12w) and dorsally obtusely arcuate in the middle in lateral view (Figure 12x). Six lateroventral preapical setae and one lateroapical seta on each side. Internal stylus of the aedeagus weakly sinuate. Spermiduct extremely short. Spermatheca bilobate, with a very small basal lobe, base of the spermatheca helical (Figure 13w).

##### Differential diagnosis.

Females are easily recognized by the spermatheca which has a helical base and the spermiduct short, not helical. When helical structures occur in the female genitalia in other species, this affects the spermiduct, and not the spermatheca.

##### DNA barcodes.

In BOLD, COI barcodes area available for the holotype, RMNH.INS.555632, and one paratype, RMNH.INS.555633, together forming the BIN ACJ9598.

##### Habitat and distribution.

Known from the type locality, on Gunung Alab, Crocker Range, Sabah, Malaysia.

##### Etymology.

Named for the type locality, Gunung Alab in the Crocker Range.

### Identification keys to genera and species

#### Key to the genera

**Table:** 

1	Mesocoxal cavities attached to one another. Protibiae without a long row of equal spines along the external side. Male first mesotarsomere dilated. Epistomal suture absent	**2**
–	Mesocoxal cavities separated by a mesoventral process. Protibiae with a long row of equal spines along the external side. Male first mesotarsomere undilated. Epistomal suture present (Ptomaphagini)	**3**
2	Elytral surface with punctuation aligned in transverse strigae. Male urite IX entire (Anemadini, Nemadina)	***Micronemadussondaicus* sp. n.**
–	Elytral surface with uniformly spaced punctuation, without transverse strigae. Male urite IX reduced (Cholevini, Catopina)	***Catopspruinosus* Schweiger**
3	Female protarsi tetramere and widely dilated (Figure 3d), approximately as wide as the apex of the protibia. Mesoventral process extremely wide and high with a flat ventral side (Figure 3c). Pronotal and elytral setae of two kinds, one long and erect, the other short and recumbent (Figure 3a) (Baryodirina)	***Baryodirushammondi* Perreau**
–	Female protarsi pentamere and undilated. Mesoventral process narrow, the ventral side sharp-edged (Ptomaphaginina)	**4**
4	Ventral spines of protibiae randomly arranged. Metaventral sutures convergent towards the central axis of the body	***Ptomaphaminus* Perreau**
–	Ventral spines of protibiae aligned along the latero-external row of equal spines, making a second, more widely-spaced row next to the external one. Metaventral sutures roughly parallel to the axis of the body	***Ptomaphaginus* Portevin**

#### Key to the species of *Ptomaphaginus*

**Table:** 

1	Body length more than 3.3 mm	**2**
–	Body length less than 3.1 mm	**4**
2	Elytra more than two times as long as the pronotum	**3**
–	Elytra not more than two times as long as the pronotum	***P.bryantioides* Schilthuizen & Perreau** (exceptionally large individuals)
3	Female: apical capsule of spermatheca annulated, U-shaped, spermiduct not extremely long, consisting of 5–6 coils (Figure 10b); male: aedeagus slightly bent ventrad and relatively flat, at the apex blunt, nearly rounded, stylet short and broad (Figure 6k–l), elytral apex without a distinct tuft of spine-like setae	***P.grandis* sp. n.**
–	Female: apical capsule of spermatheca not annulated, semicircular, spermiduct extremely long, consisting of ca. 30 coils (Figure 10c); male: aedeagus strongly curved ventrad and strongly convex, keeled, at the apex trilobate, stylet very long and thin, hair-like (Figure 7a–b), elytral apex with a distinct tuft of spine-like setae	***P.isabellarossellini* sp. n.**
4	Aedeagus with two lateral “flaps” at the apex and usually a median processus, the apex thereby appearing bilobate or trilobate (Figs 6a, c, e, g, 8a)	**5**
–	Aedeagus tip upturned or flattened, sometimes with a median processus, but not clearly bi- or trilobate (Figs 6i, m, 7c, e, g, i)	**10**
5	Habitus narrow and elongated, relatively flat. Pronotum 1.5–1.6 times as wide as long, narrowing in a more rectilinear fashion from caudal to rostral (Figure 4c)	**6**
–	Habitus broader. Pronotum 1.7–1.9 times as wide as long, narrowing in a gradual curve from caudal to rostral	**7**
6	Aedeagus apically with two long lateral flaps that jointly are more than half the width of the basal part of the aedeagus, and a long median processus. Female unknown	***P.bryanti* Jeannel**
–	Aedeagus apically with two short, triangular lateral flaps, jointly less than half the width of the basal part of the aedeagus, without a clear median processus (Figure 8a). Female unknown	***P.caroli* Schilthuizen & Perreau**
7	Aedeagus in dorsal view slender, more than two times as long as wide, at the apex with a long (longer than each of the lateral flaps), caudally pointing, median processus (Figure 6c); spermatheca without multiple, ring-shaped constrictions (Figure 10a)	***P.bryantioides* Schilthuizen & Perreau**
–	Aedeagus in dorsal view short and broad, less than two times as long as wide, if at the apex with a caudally pointing median processus, then this is shorter than each of the lateral flaps; spermatheca sometimes with ring-shaped constrictions	**8**
8	Male protarsi not dilated, less than one-third the width of the apex of the protibia; as narrow as in the female; spermatheca with multiple, ring-shaped constrictions	***P.similipes* Schilthuizen & Perreau**
–	Male protarsi moderately to strongly dilated, at least half the width of the apex of the protibia; spermatheca unknown	**9**
9	Male protarsi moderately dilated, about half as wide as the apex of the protibia; elytral apices rounded (male) or drawn out (female)	***P.kinabaluensis* Schilthuizen & Perreau**
–	Male protarsi strongly dilated, as wide as the apex of the protibia; elytral apices truncated in both sexes	***P.latimanus* Schilthuizen & Perreau**
10	Antennae short and broad, antennomeres 9 and 10 twice as long as wide; aedeagus inflated and strongly convex; spermatheca with multiple ring-shaped constrictions (Figure 10f)	***P.scaphaner* Szymczakowski**
–	Antennae slender, antennomeres 9 and 10 square or only slightly wider than long; aedeagus not inflated and highly convex; (spermatheca not known for all species)	**11**
11	Eyes reduced (each eye only one-tenth of the width of the head), wingless; female unknown	***P.burckhardti* Schilthuizen & Perreau**
–	Eyes normally developed (each eye ca. one-sixth of the width of the head), winged	**12**
12	Aedeagus in dorsal view quadrangular, two times as long as wide (Figure 7i); female unknown	***P.sabahensis* Schilthuizen & Perreau**
–	Aedeagus in dorsal view narrowed caudally, either gradually or abruptly pointed, more than two times as long as wide	**13**
13	Upturned tip of the aedeagus sharp, pointed (Figs 6m, 7e)	**14**
–	Upturned tip of the aedeagus broadly flattened and rounded (Figure 6i)	***P.anas* Schilthuizen & Perreau**
14	Apex of the aedeagus abruptly narrowed (Figure 6m); pronotum narrower than the elytra at the shoulders, caudal angles hardly drawn out (Fig. 5a); spermatheca without multiple ring-shaped constrictions (Figure 10d)	***P.louis* sp. n.**
–	Apex of the aedeagus gradually narrowed; pronotum as wide as the elytra at the shoulders, caudal angles distinctly drawn out (Figure 5b); spermatheca with multiple ring-shaped constrictions (Figure 10e)	***P.muluensis* sp. n.**

#### Key to the species of *Ptomaphaminus*

**Table:** 

1	Basal loop of the endophallus symmetric and stylus short and more strongly sinuate, located in the apical half of the median lobe (Figure 12b–d). Urite IX of the male with an extremely long and thin *spiculum gastrale*, not transversally expanded (Figure 13a–c). Aedeagus without apical hook in lateral view (Figure 12a–c). Female gonocoxites long, more than three times as long as wide (Figure 13b–d). Spermatheca weakly sclerified, the apical capsule with a set of transversal rings (Figure 13b–d). Female sutural apex of elytra spiniform	**2**
–	Basal loop of the endophallus asymmetric and stylus long and weakly sinuate, extended over most of the length of the median lobe. Anterior half of the *spiculum gastrale* of the male genital segment generally dilated into a paddle-shape (except *P.alabensis* sp. n.). Apex of aedeagus with a more or less developed hook-shaped expansion visible in lateral view. Female gonocoxites short, approximately as wide as long. Female sutural apex of elytra rounded, angular or spiniform	**3**
2	Head and discus of the elytra noticeably darker brown than the rest of the body. Male protarsi more expanded, 0.5 times as wide as the apex of protibia. Spermiduct irregular	***P.latescens* Szymczakowski**
–	Entire body light reddish brown. Male protarsi less expanded, 0.4 times as wide as the apex of protibia. Spermiduct slightly helical	***P.testaceus* sp. n.**
3	Protarsus dilated (males)	**4**
–	Protarsus undilated (females)	**14**
4	Eyes reduced (<50 ommatidia)	**5**
–	Eyes normally developed (>100 ommatidia)	**7**
5	Aedeagus long (more than 0.5 mm). Apical hook of the aedeagus long and retroverted (Figure 12e–12g)	**6**
–	Aedeagus short (~0.35 mm). Median lobe abruptly narrowed in lateral view on the apical third of its length, with a short apical hook. (Figure 12r)	***P.fagei* Perreau**
6	Apex of the aedeagus rectangular, the lateral angles sharp (Figure 12h)	***P.chapmani* Peck**
–	Lateral angles of the apex of the aedeagus rounded (Figure 12f)	***P.ater* Perreau**
7	Aedeagus long, more than 0.5 mm	**8**
–	Aedeagus short, less than 0.4 mm	**9**
8	Median lobe of the aedeagus thicker, regularly arcuate along its whole length (Figure 12k), the endophallic stylus sinuate in dorsal view (Figure 12l)	***P.hanskii* sp. n.**
–	Median lobe of the aedeagus thinner, slightly angular at the second third of its length (Figure 12i), the endophallic stylus strait in dorsal view (Figure 12j)	***P.sarawacensis* sp. n.**
9	Apical hook of the median lobe of aedeagus short (Figure 12n)	***P.nanus* sp. n.**
–	Apical hook of the median lobe of aedeagus longer	**10**
10	Apical hook of the aedeagus retroverted	**11**
–	Apical hook of the aedeagus not retroverted (Figure 12v)	***P.kinabatanganensis* sp. n.**
11	Apical hook of the median lobe of the aedeagus thick (Figure 12z). Aedeagus extremely small (0.22 mm) (Figure 12y–z)	***P.microphallus* sp. n.**
–	Apical hook of the median lobe of the aedeagus thin	**12**
12	Apical hook of the median lobe of aedeagus angular (Figure 12p)	***P.marshalli* sp. n.**
–	Apical hook of the median lobe of aedeagus roundish	**13**
13	*Spiculum gastrale* of the male genital segment slightly expanded in the anterior part (Figure 13o). Median lobe of aedeagus widely rounded at apex in dorsal view (Figure 12s)	***P.layangensis* sp. n.**
–	*Spiculum gastrale* of the male genital segment not expanded in the anterior part (Figure 13v). Median lobe of aedeagus more or less triangularly narrowed at the end in dorsal view, the apex narrowly rounded (Figure 12w)	***P.alabensis* sp. n.**
14	Sutural apex of elytra sexually dimorphic, rounded in males, angular or spiniform in females (Figure 14c–e)	**15**
–	Sutural apex of elytra not sexually dimorphic, rounded in females as in males (Figure 14b)	**19**
15	Eyes reduced (<50 ommatidia). Sutural apex of elytra spiniform (Figure 14d–e)	**16**
–	Eyes normally developed (>80 ommatidia). Sutural apex of elytra simply angular (Figure 14c)	**18**
16	Spermiduct not helical (Figure 13s)	***P.chapmani* Peck**
–	Spermiduct helical	**17**
17	Spermatheca with an apical sclerotised plate (Figure 13f)	***P.ater* Perreau**
–	Spermatheca without apical plate (Figure 13l)	***P.fagei* Perreau**
18	Spermiduct not helical (Figure 13n)	***P.kinabatanganensis* sp. n.**
–	Spermiduct membranous, vaguely helical near the base of the spermatheca (Figure 13h–j)	***P.hanskii* sp. n. and *P.sarawacensis* sp. n.**
19	Spermiduct not helical	**20**
–	Spermiduct helical	**21**
20	Terminal lobe of spermatheca regularly narrowed, the apex conical (Figure 13y)	***P.marshalli* sp. n.**
–	Apex of spermatheca rounded, base of the spermatheca helical (but not the spermiduct) (Figure 13w)	***P.alabensis* sp. n.**
21	Very small size, <1.3 mm. Spermiduct with many coils tightly compacted (Figure 13u)	***P.nanus* sp. n.**
–	Normal size, >1.5 mm. Spermiduct with coils regularly spaced, not tightly packed (Figure 13p)	***P.layangensis* sp. n.**

**Figure 15.**
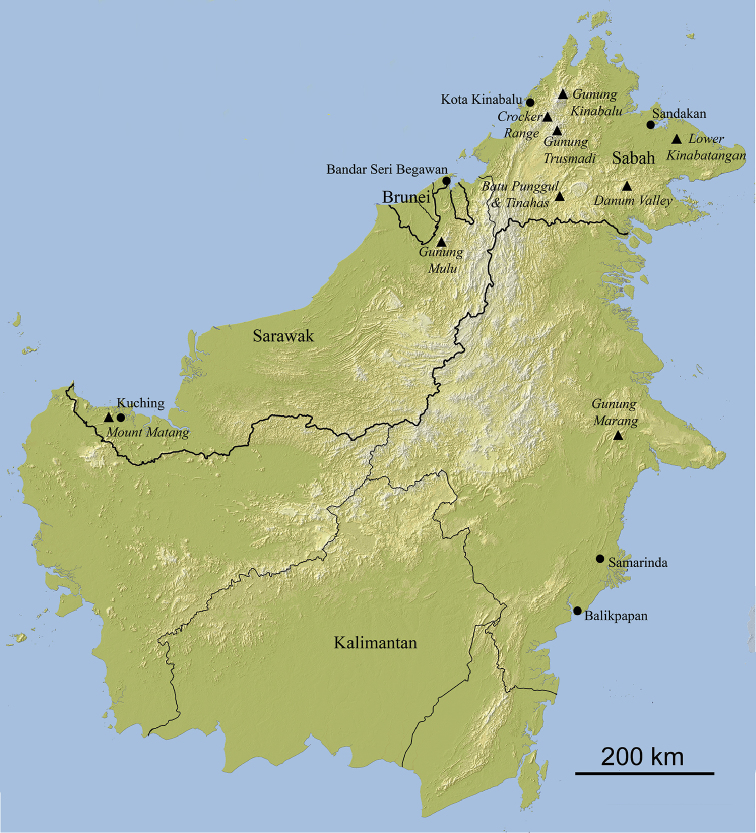
Map of Borneo, with the areas that feature in this paper indicated.

## Supplementary Material

XML Treatment for Micronemadus

XML Treatment for Micronemadus sondaicus

XML Treatment for Catops

XML Treatment for Catops pruinosus

XML Treatment for Baryodirus

XML Treatment for Baryodirus hammondi

XML Treatment for Ptomaphaginus

XML Treatment for Ptomaphaginus anas

XML Treatment for Ptomaphaginus bryanti

XML Treatment for Ptomaphaginus bryantioides

XML Treatment for Ptomaphaginus burckhardti

XML Treatment for Ptomaphaginus caroli

XML Treatment for Ptomaphaginus grandis

XML Treatment for Ptomaphaginus isabellarossellini

XML Treatment for Ptomaphaginus kinabaluensis

XML Treatment for Ptomaphaginus latimanus

XML Treatment for Ptomaphaginus louis

XML Treatment for Ptomaphaginus muluensis

XML Treatment for Ptomaphaginus sabahensis

XML Treatment for Ptomaphaginus scaphaner

XML Treatment for Ptomaphaginus similipes

XML Treatment for Ptomaphaminus

XML Treatment for Ptomaphaminus latescens

XML Treatment for Ptomaphaminus testaceus

XML Treatment for Ptomaphaminus ater

XML Treatment for Ptomaphaminus kinabatanganensis

XML Treatment for Ptomaphaminus chapmani

XML Treatment for Ptomaphaminus fagei

XML Treatment for Ptomaphaminus nanus

XML Treatment for Ptomaphaminus marshalli

XML Treatment for Ptomaphaminus hanskii

XML Treatment for Ptomaphaminus sarawacensis

XML Treatment for Ptomaphaminus layangensis

XML Treatment for Ptomaphaminus microphallus

XML Treatment for Ptomaphaminus alabensis
